# Mesothelioma Tumor Cells Modulate Dendritic Cell Lipid Content, Phenotype and Function

**DOI:** 10.1371/journal.pone.0123563

**Published:** 2015-04-17

**Authors:** Joanne K. Gardner, Cyril D. S. Mamotte, Priya Patel, Teong Ling Yeoh, Connie Jackaman, Delia J. Nelson

**Affiliations:** 1 Immunology and Cancer Group, School of Biomedical Sciences, Curtin University, Perth, Western Australia, Australia; 2 CHIRI Biosciences Research Precinct, Curtin University, Perth, Western Australia, Australia; University of Pittsburgh, UNITED STATES

## Abstract

Dendritic cells (DCs) play an important role in the generation of anti-cancer immune responses, however there is evidence that DCs in cancer patients are dysfunctional. Lipid accumulation driven by tumor-derived factors has recently been shown to contribute to DC dysfunction in several human cancers, but has not yet been examined in mesothelioma. This study investigated if mesothelioma tumor cells and/or their secreted factors promote increases in DC lipid content and modulate DC function. Human monocyte-derived DCs (MoDCs) were exposed to human mesothelioma tumor cells and tumor-derived factors in the presence or absence of lipoproteins. The data showed that immature MoDCs exposed to mesothelioma cells or factors contained increased lipid levels relative to control DCs. Lipid accumulation was associated with reduced antigen processing ability (measured using a DQ OVA assay), upregulation of the co-stimulatory molecule, CD86, and production of the tolerogenic cytokine, IL-10. Increases in DC lipid content were further enhanced by co-exposure to mesothelioma-derived factors and triglyceride-rich lipoproteins, but not low-density lipoproteins. In vivo studies using a murine mesothelioma model showed that the lipid content of tumor-infiltrating CD4^+^CD8α^-^ DCs, CD4^-^CD8α^-^ DCs DCs and plasmacytoid DCs increased with tumor progression. Moreover, increasing tumor burden was associated with reduced proliferation of tumor-antigen-specific CD8^+^ T cells in tumor-draining lymph nodes. This study shows that mesothelioma promotes DC lipid acquisition, which is associated with altered activation status and reduced capacity to process and present antigens, which may impair the ability of DCs to generate effective anti mesothelioma T cell responses.

## Introduction

Dendritic cells (DCs) are important mediators of anti-tumor immune responses. Under ideal conditions DCs present tumor-associated antigens to activate tumor-specific cytotoxic CD8^+^ T cells, which then migrate into tumors to lyse tumor cells. However, there is evidence that DCs in cancer patients are dysfunctional, preventing the generation of effective anti-tumor responses [1]. Tumor-derived factors such as vascular endothelial growth factor (VEGF) [2–4], transforming growth factor (TGF)-β [5], interleukin (IL)-6 [6] and IL-10 [7,8] cause DC dysfunction by interfering with the DC lifecycle, including differentiation [8,9], maturation [4,10] and migration [11], thereby impairing the ability of DCs to activate T cells. Recently, lipid acquisition driven by tumor-derived factors has been shown to contribute to DC dysfunction in human cancers of the head-and-neck, lung and kidney, and in murine lymphoma, colon and mammary adenocarcinomas [12]. Increased intracellular DC triglyceride content was associated with reduced ability of DCs to process antigens and stimulate T cell proliferation [12].

Tumor-driven DC lipid accumulation and associated effects on DC function had not, until now, been investigated in mesothelioma, an aggressive cancer of the pleural membranes [13]. This study aimed to determine if mesothelioma tumor cells and/or their products impair DC function by driving DC lipid acquisition. An in vitro model of human monocyte-derived DCs (MoDCs) was used to investigate the effect of human mesothelioma cells on the capacity of CD14^+^ monocytes to differentiate into immature DCs, as well as the capacity of these immature DCs to respond appropriately to maturation stimuli. Effects of mesothelioma cells/factors on DC lipid content, surface phenotype, antigen processing function and cytokine production were investigated. A murine mesothelioma model (AE17) was used to study the in vivo relationship between tumor burden, lipid content of DC subsets and tumor antigen presentation to CD8^+^ T cells in the tumor microenvironment and lymphoid organs.

## Materials and Methods

### Mice

Female C57BL/6J mice aged 6–8 weeks were obtained from the Animal Resources Centre (ARC; Perth, Australia). The OT-1 (H-2^b^) TCR transgenic mouse line, expressing a CD8^+^ TCR recognizing the dominant H-2^b^ restricted ovalbumin epitope, SIINFEKL, was kindly supplied by Professor I. Frazer and Dr. R. Steptoe (University of Queensland, Australia) and bred at the ARC. All mice were maintained under specific pathogen-free conditions in the Curtin University animal facility. All mouse experiments were performed according to the Australian Code of Practice for the care and use of animals for scientific purposes and the Curtin University Animal Ethics Committee (AEC) specifically approved this study (approval numbers AEC-2011-01, AEC-2011-01A and AEC-2011-16).

### Human ethics approval

Curtin University, Sir Charles Gairdner Hospital and the Mount Hospital Human Ethics Committees approved the human studies (approval numbers HR 68/2008, 2008–041 and EC50.1 respectively). Written consent was obtained from all study participants.

### Cell lines and tumor cell-conditioned media

JU77 is a human mesothelioma cell line established from the pleural effusion of a patient with confirmed disease diagnosis [14]. Met5A is a non-malignant transformed human mesothelial cell line established by transfecting normal pleural mesothelial cells with Simian virus 40 DNA [15]. AE17 is a murine mesothelioma cell line derived from the peritoneal cavity of a C57BL/6J mouse injected with asbestos fibres, as previously described [16]. AE17sOVA was developed by transfecting the AE17 parental cell line with cDNA coding for secretory ovalbumin (OVA), hence ovalbumin, which contains the SIINFEKL peptide, functions as a marker tumor antigen, as previously described [16]. All cell lines were maintained in complete medium, which consisted of RPMI 1640 (Invitrogen, California, USA) supplemented with 10% FCS (ThermoScientific, Scoresby, Australia), 50 mg/L gentamicin (Pharmacia and Upjohn, Perth, Australia), 60 mg/L benzylpenicillin (CSL Ltd, Parkville, Australia), 2 mM L-glutamax (Invitrogen, Grand Island, USA) and 0.05 mM 2-mercaptoethanol (Sigma-Aldrich, Sydney, Australia). The transfected AE17sOVA tumor cell line was maintained in complete medium supplemented with 400 μg/L of the neomycin analogue G418 (Geneticin; Life Technologies, Grand Island, USA). All cells were cultured at 37°C in a 5% CO_2_ atmosphere.

For the generation of tumor cell-conditioned media, JU77 and AE17 cell lines were cultured in serum-free medium (Invitrogen). After 48–72 hours, conditioned media from tumor cell cultures were centrifuged and cell-free supernatants collected and stored at -80°C until use.

### In vitro generation of human monocyte-derived DCs (MoDCs)

Peripheral whole blood (50 ml) was collected from 6 female and 2 male (ages ranging from 22 to 35 years) healthy human volunteers into EDTA vacutainer tubes (Beckton Dickinson (BD), San Jose, USA). Peripheral blood mononuclear cells (PBMCs) were isolated by diluting blood 1:2 in PBS containing 2 mM EDTA (Sigma-Aldrich), followed by density gradient centrifugation on Ficoll-Paque^TM^ Plus (GE Healthcare, Sydney, Australia). PBMCs were washed three times in PBS containing 2 mM EDTA, and resuspended in complete medium. To obtain monocytes, PBMCs were incubated in tissue culture flasks for 2 hours at 37°C then non-adherent lymphocytes were removed. Adherent monocytes were differentiated into immature MoDCs by culturing for 7 days in complete medium supplemented with 80 ng/ml human granulocyte-macrophage colony-stimulating factor (GM-CSF, eBiosciences, San Diego, USA), 10 ng/ml recombinant human IL-4 (eBiosciences) and 10 μg/ml Polymixin B (Sigma-Aldrich). At day 7, immature DCs were collected for analysis, or used to generate mature DCs by culturing in complete medium containing 80 ng/ml GM-CSF, 10 ng/ml IL-4 and 10 μg/ml LPS (Sigma-Aldrich) for 2 days. Cultures were supplemented with growth factors at days 4 and/or 7 and maintained at 37°C in 5% CO_2_. For lipid-lowering studies, 5-tetradecycloxy-2-furoic acid (TOFA; Enzo Life Sciences, New York, USA) was added to MoDC cultures at a final concentration of 5 μg/ml for days 4–7. Control cultures had the equivalent volume of DMSO, the TOFA diluent control, added for days 4–7.

### DQ-ovalbumin assay

DQ-ovalbumin (DQ-OVA; Invitrogen), a self-quenched conjugate of ovalbumin that fluoresces upon proteolytic degradation [17], was used to measure DC antigen uptake and processing. DCs (5 x 10^4^) were suspended in 100 μl of complete medium containing 10 μg/ml of DQ-OVA and incubated at 4°C or 37°C for 1 hour. Controls consisting of 5 x 10^4^ DCs without DQ-OVA were also included. Cells were washed once with 200 μl of PBS and resuspended in 100 μl of FACS buffer (PBS containing 2% FCS and 1% BSA). The level of DQ-OVA fluorescence (mean fluorescence intensity; MFI) of the DCs was analyzed by flow cytometry. To calculate the level of DQ-OVA uptake and processing, the following formula was used: DQ-OVA processing MFI = [MFI at 37°C with DQ-OVA—MFI at 37°C without DQ-OVA]–[MFI at 4°C with DQ-OVA—MFI at 4°C without DQ-OVA].

### Allogeneic mixed lymphocyte reaction (MLR)

Allogeneic T cells were labelled with carboxyfluorescein diacetate succinimidyl ester (CFSE; Invitrogen) a fluorescent dye that binds permanently to cell membranes [18]. 2 x 10^5^ CFSE-labelled T cells were co-cultured with varying ratios of DCs for 8 days in 200 μl of complete medium at 37°C. Positive controls were T cells cultured with 5 μg/ml Con A (Sigma-Aldrich). At day 8, cells were stained with antibodies to identify CD4^+^ and CD8^+^ T cells by flow cytometry. As T cells proliferate, CFSE segregates equally between each daughter population; in flow cytometric analysis each round of proliferation is seen as sequential halving of CFSE staining intensity. The percent of T cell proliferation was calculated based on the loss of staining intensity of the parent peak.

### Cytokine measurement

Tumor necrosis factor-alpha (TNF-α), IL-12p70, IL-10, interferon gamma (IFN-γ) and VEGF concentrations were measured using a cytometric bead array (CBA; BD) in accordance with the manufacturer’s instructions. Briefly, samples and cytokine standards were incubated with cytokine capture beads for 1 hour at room temperature in a 96-well plate, followed by incubation with PE-conjugated detection antibodies for 2 hours at room temperature, washing with 150 μl of wash buffer per well and centrifugation at 200 x *g* for 5 minutes. Samples/standards were resuspended in 100 μl of wash buffer and analysis performed on a FACSCanto II using FACSDiva software (both from BD).

### Lipoprotein isolation

A single step density gradient ultracentrifugation was used to isolate the triglyceride (TG)-rich (*d* < 1.0063 g/ml) and low-density lipoprotein (LDL; 1.019 < *d* > 1.063 g/ml) fractions from the sera of healthy human volunteers, as we have previously described [19]. Briefly, 30 ml of blood collected into serum separation tubes (SST; BD) was allowed to clot for 1 hour at room temperature. Serum was separated by centrifugation at 3000 x *g* for 30 minutes and adjusted to a density of 1.07 g/ml using NaCl (Sigma-Aldrich). Density-adjusted serum, 4 ml, was loaded into a 13.5 ml thin-walled polycarbonate tube (Beckman Coulter, Brea, USA) and overlaid with a discontinuous gradient constructed by sequentially layering 2 ml each of solutions of *d* = 1.063, 1.04, 1.02 g/ml, followed by 2 ml MilliQ water. Density solutions were prepared using NaCl and 1 mM EDTA (Sigma-Aldrich) and checked using a digital density meter (DA-110, Kyoto Electronics, Kyoto, Japan) prior to use. Samples were centrifuged for 18 hours at 40,000 rpm and 20°C using a Sorvall WX Ultra Centrifuge and TH-641 swinging bucket rotor (ThermoScientific).

Following centrifugation, TG-rich lipoproteins were collected in the top 1 ml fraction, and LDL in the 1 ml fraction corresponding to the visible yellow band. Fractions were dialyzed against three changes of 1.5 L PBS containing 1 mM EDTA for 24 hours at 4°C. The protein content was assayed using a modified Lowry assay (Bio Rad Laboratories, Hercules, USA), and lipid composition determined using colorimetric cholesterol and triglyceride assays (Randox Laboratories, Antrim, UK). Fractions were further characterized using agarose gel electrophoresis, stained with either a lipid stain (Sudan Black) or a protein stain. All fractions were filter-sterilized using a 0.2 μm filter (Corning Inc., New York, USA) and used within 24 hours of isolation. For addition to MoDC cultures, TG-rich lipoproteins were used at a final TG concentration of 0.1 mM and LDL was used at a final cholesterol concentration of 0.5 mM.

### In vivo murine tumor growth, treatments and tissue collection

At day 0, mice were injected subcutaneously in the right flank with 5 x 10^5^ AE17 or AE17sOVA tumor cells per mouse in 100 μl of PBS and tumors measured regularly using microcallipers. For lipid-lowering studies, on day 1 following AE17 tumor cell inoculation, mice were anaesthetized using methoxyflurane and surgically implanted (subcutaneously, behind the scapulae) with osmotic pumps (ALZET model 1002, Durect, California, USA), containing TOFA or an equivalent amount of the TOFA diluent control, DMSO. Mice received a dose of 1 μg TOFA per hour, continuously for 14 days. Mice were euthanized when tumors reached 100 mm^2^, the maximum allowable size as per ethics committee approval. At end point, tumors, spleens, tumor-draining lymph nodes and non-draining lymph nodes were collected and disaggregated into single cell suspensions by gentle dispersion between two frosted glass slides.

### In vitro generation of murine bone marrow-derived DCs (BMDCs)

DCs were generated from murine bone marrow haemopoietic stem cells using a procedure adapted from Lutz et al. [20]. Briefly, femurs and tibiae from healthy mice were collected, and bone marrow flushed using complete medium in a 0.5 ml insulin syringe. Bone marrow haemopoietic stem cells were seeded at 1 x 10^6^ cells per bacteriological grade Optilux petri dish (BD). Cultures were supplemented with 20 ng/ml recombinant mouse GM-CSF (eBiosciences), 20 ng/ml IL-4 (eBiosciences) and 10 μg/ml Polymixin B (Sigma-Aldrich) on days 0, 3, 6, 8 and 10. At day 10, BMDCs were collected for analysis, or further matured by culturing with 20 ng/ml GM-CSF, 20 ng/ml IL-4 and 10 μg/ml LPS (Sigma-Aldrich) for 2 days.

### Flow cytometry: phenotyping and staining with BODIPY 493/503 for lipids

Human MoDCs were stained with fluorescently labelled antibodies directed against human CD11c (clone B-Iy6), CD1a (clone HI149), HLA-DR (clone L243), CD86 (clone 2331(FUN-1)) and CD80 (clone 2D10), all from BD Pharmingen, San Jose, USA. Murine DCs were stained with antibodies against murine CD11c (clone N418; Biolegend, San Diego, USA); anti-CD4 (clone RM4-5, Caltag, Towcester, UK) and anti-CD8α, anti-B220 and anti-Gr1 (clones 53–6.7, RA3-6B2, RB6.8C5 respectively; BD Pharmingen).

Cells were incubated with antibodies for 30 minutes at 4°C in the dark, washed using PBS and resuspended in 50 μl of 5 μg/ml BODIPY 493/503 (Invitrogen) for 15 minutes at 4°C, washed and resuspended in 100 μl of FACS buffer. Analysis was performed on a FACSCanto II using FACSDiva software (BD) or FlowJo software (TreeStar, Oregon, USA).

### Lyons Parish assay: in vivo analysis of tumor antigen cross-presentation

The Lyons Parish assay was used to assess in vivo changes in tumor antigen presentation levels using adoptively transferred tumor-specific CD8^+^ T cells from OT-1 mice. To do this, cells were labelled with CFSE as previously described [16]. The cells were resuspended in 20 ml RPMI at 10^7^ cells/ml and incubated with 1 μl of CFSE stock solution (5 mM in DMSO) for 10 minutes at room temperature. Cells were washed through FCS four times and PBS twice, and 10^7^ cells intravenously injected into each recipient mouse. CFSE-labelled cells were recovered from secondary lymphoid organs 3 days post adoptive transfer and analyzed for CD8 expression by FACS analysis.

### Immunofluorescence

Tissue samples were embedded in Tissue-Tek O.C.T. freezing medium (ProScitech, Kirwan, Australia) and placed immediately at -80°C. Sections (8 μm) were cut on a Leica HM 550 microtome cryostat, placed on poly-L lysine (Sigma-Aldrich) coated glass slides (ProScitech) and stored at -20°C until use. Slides were brought to room temperature, sections were fixed in 4% paraformaldehyde (Sigma-Aldrich) for 15 minutes at room temperature, washed once in PBS for 5 minutes, then blocked and permeabilised in PBS/10% FCS/1% BSA/0.1% saponin for 1 hour at room temperature. Sections were then incubated with 1 μg/ml BODIPY diluted in PBS/10% FCS/1% BSA in the dark for 1 hour at room temperature. Slides were then washed 3 times in PBS (5 minutes per wash), mounted in Citifluor^TM^ anti-fadent (ProScitech) and visualised on an Olympus IX51 microscope (Olympus, Centre Valley, USA) with cellSens software (Olympus). Images were compiled using Adobe Photoshop CS5 extended software (Adobe, San Jose, USA).

### Data analysis

Statistical significance was calculated using Student’s *t*-test and Mann–Whitney *U*-test on the program GraphPad PRISM 4 (GraphPad Software Inc, California, USA). *P* values of < 0.05 were considered statistically significant.

## Results

### Mesothelioma tumor cells promote lipid acquisition by immature monocyte-derived DCs

**Fig 1:**
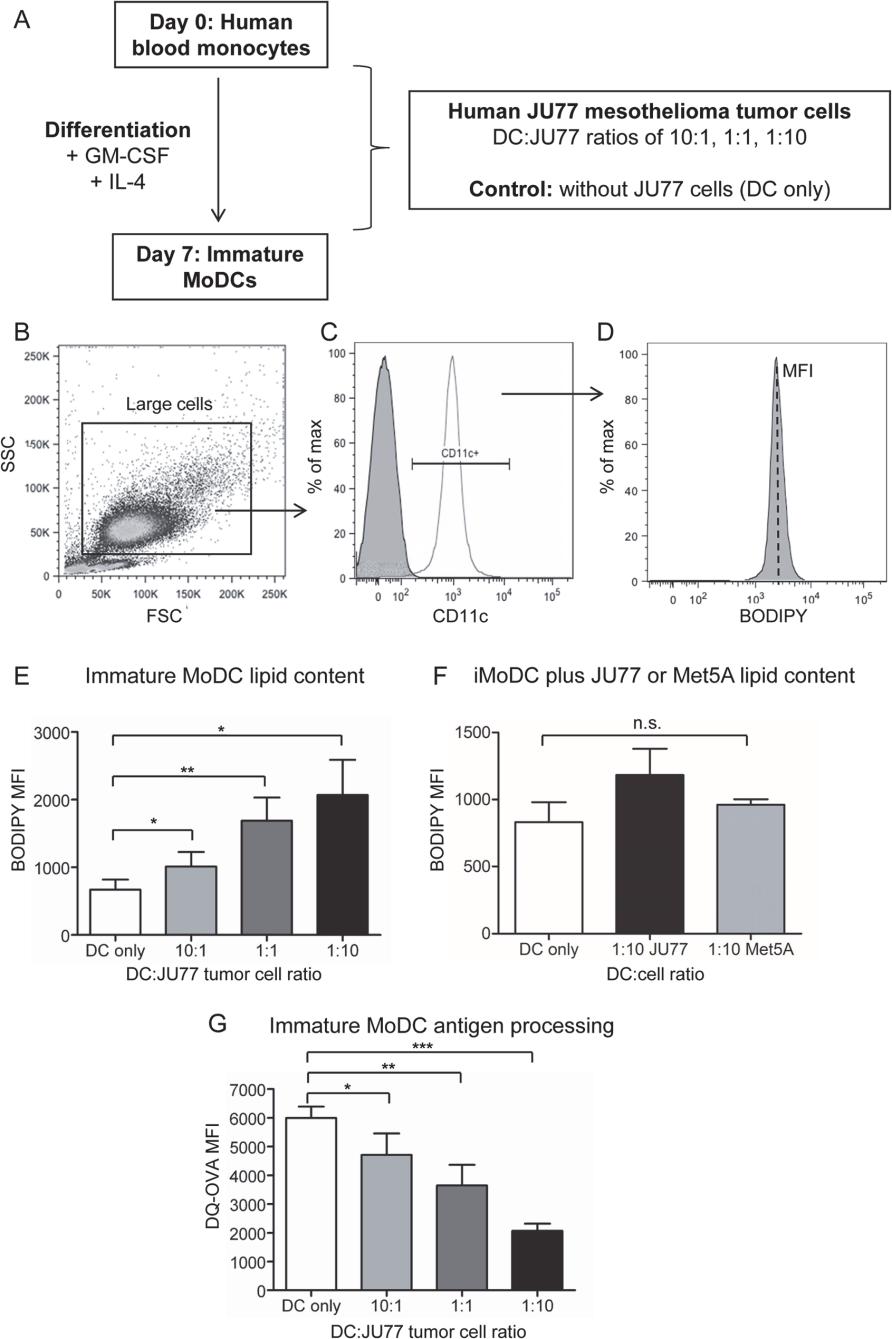
Mesothelioma tumor cells modulate human MoDC lipid content and function. Human blood monocytes cultured with GM-CSF and IL-4 were exposed to varying ratios of JU77 mesothelioma tumor cells (A). At day 7, iMoDCs stained for CD11c expression and lipid levels using BODIPY were analysed by flow cytometry. Large cells were first gated (B) and CD11c^+^ DCs identified (unfilled line), relative to the isotype control (shaded area; C). The BODIPY mean fluorescence intensity (MFI) of CD11c^+^ DCs is proportional to intracellular lipid content (D). Pooled data shows lipid levels of iMoDCs co-cultured with varying ratios of JU77 tumor cells (E). During differentiation, DCs were also exposed to Met5A cells (at a ratio of 1DC: 10Met5A cells), and DC lipid content measured as BODIPY MFI (F). The DQ-OVA assay was used to measure the antigen processing ability of iMoDCs exposed to JU77 tumor cells (G). Data in (E) and (G) is from 6 individuals and data in (F) is from 2 individuals; all data shown as mean ± SEM. * = p < 0.05; ** = p < 0.005; *** = p < 0.0005.

A recent study showed that factors derived from several human and murine tumors promoted increased intracellular lipid levels in DCs, and that these lipid-laden DCs were functionally impaired [12]. Until now, no similar studies had been performed in mesothelioma. The first series of experiments examined the effect of human JU77 mesothelioma cells on the process of blood monocytes differentiating into immature human MoDCs (iMoDCs) using co-culture studies (Fig 1A). As a normal control, iMoDCs were also co-cultured with non-malignant Met5A cells, a human pleural mesothelial cell line. Following co-culture, the lipophilic fluorescent dye BODIPY 493/503 was used to measure iMoDC (Fig 1B–1D) and JU77 tumor cell lipid content (S1A and S1B Fig). Immature MoDCs exposed to JU77 tumor cells had significantly increased lipid content relative to DCs only; shown as mean fluorescence intensity (MFI; Fig 1E). Exposure to JU77 cells also promoted a greater increase in iMoDC lipid content compared to Met5A cells (Fig 1F). The lipid content of JU77 tumor cells co-cultured with iMoDCs did not change significantly relative to the JU77 only control (S1 C Fig).

### Mesothelioma tumor cells reduce the capacity of MoDCs to process antigen

Immature DCs are characterized by their ability to take up and process antigens from their surrounding environment. The DQ-OVA assay was used to investigate if mesothelioma-induced lipid accumulation was associated with changes in the capacity of iMoDCs to process antigens. Immature MoDCs exposed to tumor cells demonstrated reduced DQ-OVA fluorescence (measured by MFI), consistent with reduced antigen processing, relative to DCs only (Fig 1G). Exposure to Met5A cells did not alter iMoDC antigen processing ability, relative to DCs only (S2A Fig). Taken together, these results show that DCs exposed to mesothelioma tumor cells, but not Met5A mesothelial cells, have increased lipid content which is associated with decreased antigen processing.

### Mesothelioma cells promote phenotypic maturation of immature MoDCs

**Fig 2:**
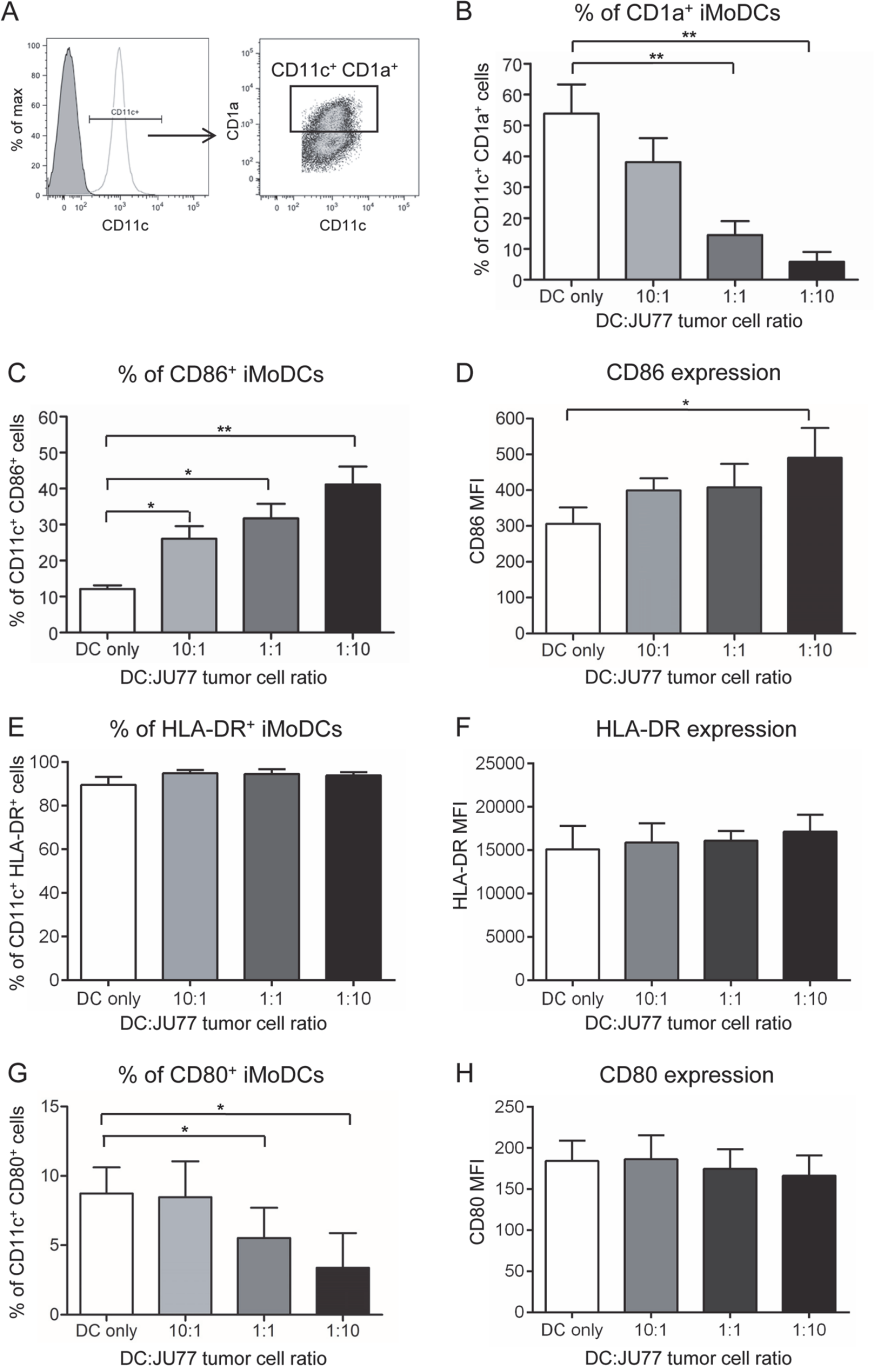
Mesothelioma tumor cells promote partial DC maturation. Differentiating iMoDCs exposed to varying ratios of JU77 cells were stained for CD11c and maturation markers for flow cytometric analysis. CD11c^+^ DCs were identified as described in Fig 1. Expression of each surface marker was analyzed on gated CD11c^+^ cells; representative gating strategy is shown (A). Pooled data of the percent of iMoDCs expressing CD1a (B), CD86 (C), HLA-DR (E) and CD80 (G) and surface expression levels (shown as MFIs) of CD86 (D), HLA-DR (F) and CD80 (H) on iMoDCs is from 6 individuals and shown as mean ± SEM. * = p < 0.05; ** = p < 0.005.

Loss of the capacity to process antigen is associated with DC maturation. Thus, iMoDCs from the above experiment were assessed for expression of surface molecules associated with maturation; representative gating strategy is shown in Fig 2A. Expression of CD1a, a molecule involved in the presentation of lipid antigens to T cells, was examined. As the proportion of JU77 mesothelioma cells increased, the percentage of CD11c^+^ iMoDCs expressing CD1a decreased (Fig 2B). These data suggest impaired capacity to present lipid antigens to T cells in lipid-laden DCs however, reduced CD1a has also been associated with DC maturation [21].

Expression of HLA-DR (an MHC class II molecule involved in the presentation of antigens to CD4^+^ T cells) and expression of CD80 and CD86 (co-stimulatory molecules required for T cell activation) were also measured. These molecules are expressed at low levels on immature DCs, but are upregulated during DC maturation. Significant increases in the percent of CD86^+^ iMoDCs were observed at all ratios of DC:JU77 cells (Fig 2C). Similarly, the surface expression levels (shown as MFI) of CD86 on iMoDCs were significantly increased at a ratio of 1 DC:10 JU77 cells (Fig 2D). Exposure to tumor cells did not significantly modulate the percent of HLA-DR^+^ iMoDCs (Fig 2E) or HLA-DR surface expression levels on iMoDCs (Fig 2F). The percent of CD80^+^ iMoDCs was significantly reduced at DC:JU77 ratios of 1:1 and 1:10 (Fig 2G) however, CD80 surface expression levels on iMoDCs did not change (Fig 2H). The changes to iMoDC surface phenotype are likely to be tumor-driven, as exposure to Met5A cells did not alter the percent of CD1a^+^, CD86^+^ or CD80^+^ iMoDCs (S2B—S2D Fig), nor did it alter surface expression levels (MFIs) of CD86 or CD80 on iMoDCs, relative to DCs only (S2E and S2F Fig). The upregulation of CD86 and downregulation of CD1a expression suggests that mesothelioma tumor cells induce partial iMoDC maturation.

To investigate whether mesothelioma cells also induced functional maturation of iMoDCs, the ability of iMoDCs to stimulate T cell proliferation was assessed using the allogeneic mixed lymphocyte reaction (MLR) assay. Exposure to tumor cells did not interfere with the ability of iMoDCs to stimulate CD4^+^ or CD8^+^ T cell proliferation (S3A and S3B Fig). Taken together, these results suggest that mesothelioma tumor cells induce inappropriate maturation of iMoDCs.

### Mesothelioma tumor cells promote production of tolerogenic cytokines by immature MoDCs

**Fig 3:**
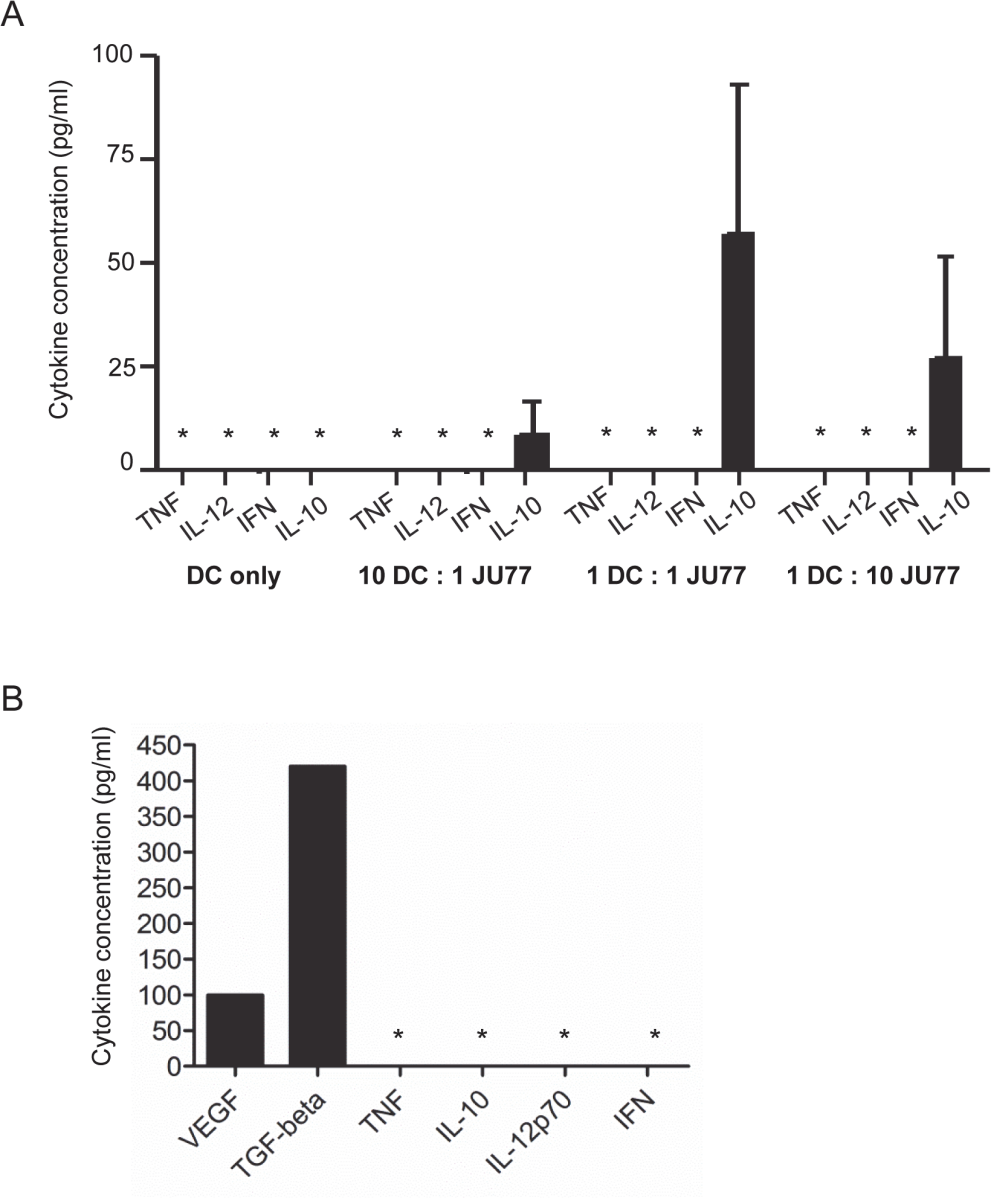
Mesothelioma cells promote production of tolerogenic cytokines by iMoDCs. Cytokine concentrations were measured in conditioned medium collected from co-cultures of immature MoDCs and JU77 tumor cells using a cytometric bead array; pooled data from 3 individuals is expressed as mean ± SEM (A). Cytokine concentrations were also measured in conditioned medium collected from cultured JU77 tumor cells; data is from one experiment (B). * = concentration below detection limit of assay.

The effect of mesothelioma cells on iMoDC cytokine production was assessed using conditioned media collected from the above experiments. The pro-inflammatory cytokines TNF-α, IL-12p70 and IFN-γ were not detected in the culture media from DC and JU77 tumor cell co-cultures (Fig 3A). In contrast, the anti-inflammatory cytokine IL-10 was detected at all ratios of DCs:JU77 tumor cells (Fig 3A). JU77 cells alone did not produce IL-10 (Fig 3B), suggesting that the source of IL-10 was the iMoDCs. These data suggest that mesothelioma cells induce tolerogenic iMoDCs that disable anti-mesothelioma immune responses.

### Modulation of MoDC differentiation is mediated by mesothelioma-derived factors

**Fig 4:**
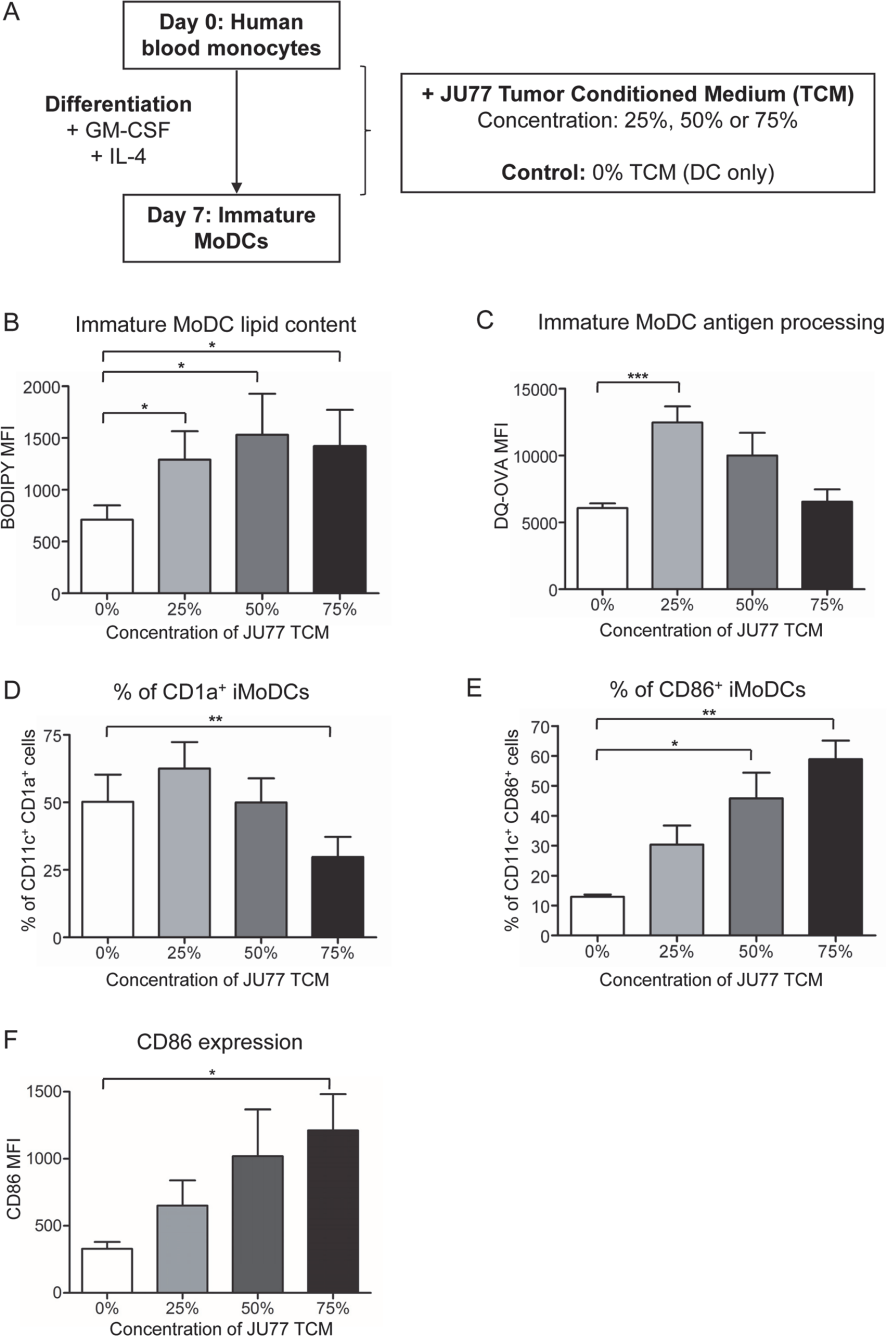
Mesothelioma-derived soluble factors modulate immature MoDC lipid content. Different concentrations of JU77 tumor cell-conditioned medium (TCM) were included in the human DC differentiation protocol (A) and lipid levels measured using BODIPY (shown as MFI; B). The antigen processing capacity of iMoDCs exposed to JU77 TCM was assessed using the DQ-OVA assay and is shown as DQ-OVA MFI (C). Expression of DC maturation markers was also measured. Pooled data shows the percent of iMoDCs positive for CD1a (D) and CD86 (E), and surface expression levels of CD86 on iMoDCs (shown as MFI; F) after exposure to varying concentrations of TCM. Pooled data in (B-F) is from 6 individuals. All data is shown as mean ± SEM. * = p < 0.05; ** = p < 0.005; *** = p < 0.0005.

The modulation of MoDC differentiation by tumor cells could be mediated by cell-to-cell contact and/or factors secreted by tumor cells. Thus, the next series of studies investigated whether mesothelioma-derived factors could also modulate MoDC lipid content, function, phenotype and cytokine production. Human monocytes were cultured with varying concentrations of tumor cell-conditioned medium (TCM) collected from cultured JU77 cells (Fig 4A). Immature MoDCs exposed to JU77 TCM demonstrated significantly higher lipid levels relative to iMoDCs cultured without TCM (Fig 4B). These data suggest that tumor-derived factors present in JU77 TCM modulate iMoDC lipid accumulation.

In contrast to iMoDCs co-cultured with tumor cells, iMoDCs exposed to TCM not only retained but improved their ability to take up and process antigen (Fig 4C). This suggests that modulation of this iMoDC function requires close contact with tumor cells. The percent of iMoDCs expressing CD1a significantly decreased after exposure to 75% JU77 TCM (Fig 4D). The percent of CD86^+^ iMoDCs increased at all concentrations of TCM, reaching statistical significance at 50% and 75% JU77 TCM (Fig 4E), and CD86 surface expression levels were significantly increased at 75% JU77 TCM (Fig 4F). Exposure to JU77 TCM did not alter the percent of HLA-DR^+^ (S4A Fig) or CD80^+^ iMoDCs (S4C Fig) or the surface expression levels of HLA-DR (S4B Fig) or CD80 (S4D Fig) on iMoDCs. These data suggest that mesothelioma-derived factors in TCM induce partial maturation of iMoDCs, and that relatively high levels of tumor-derived factors are required for these effects. The cytokines IFN-γ, IL-12p70, TNF-α and IL-10 were not detected in culture media from iMoDCs exposed to JU77 TCM. Exposure to TCM also did not affect the capacity of iMoDCs to stimulate CD4^+^ and CD8^+^ T cell proliferation (S3C and S3D Fig).

**Fig 5:**
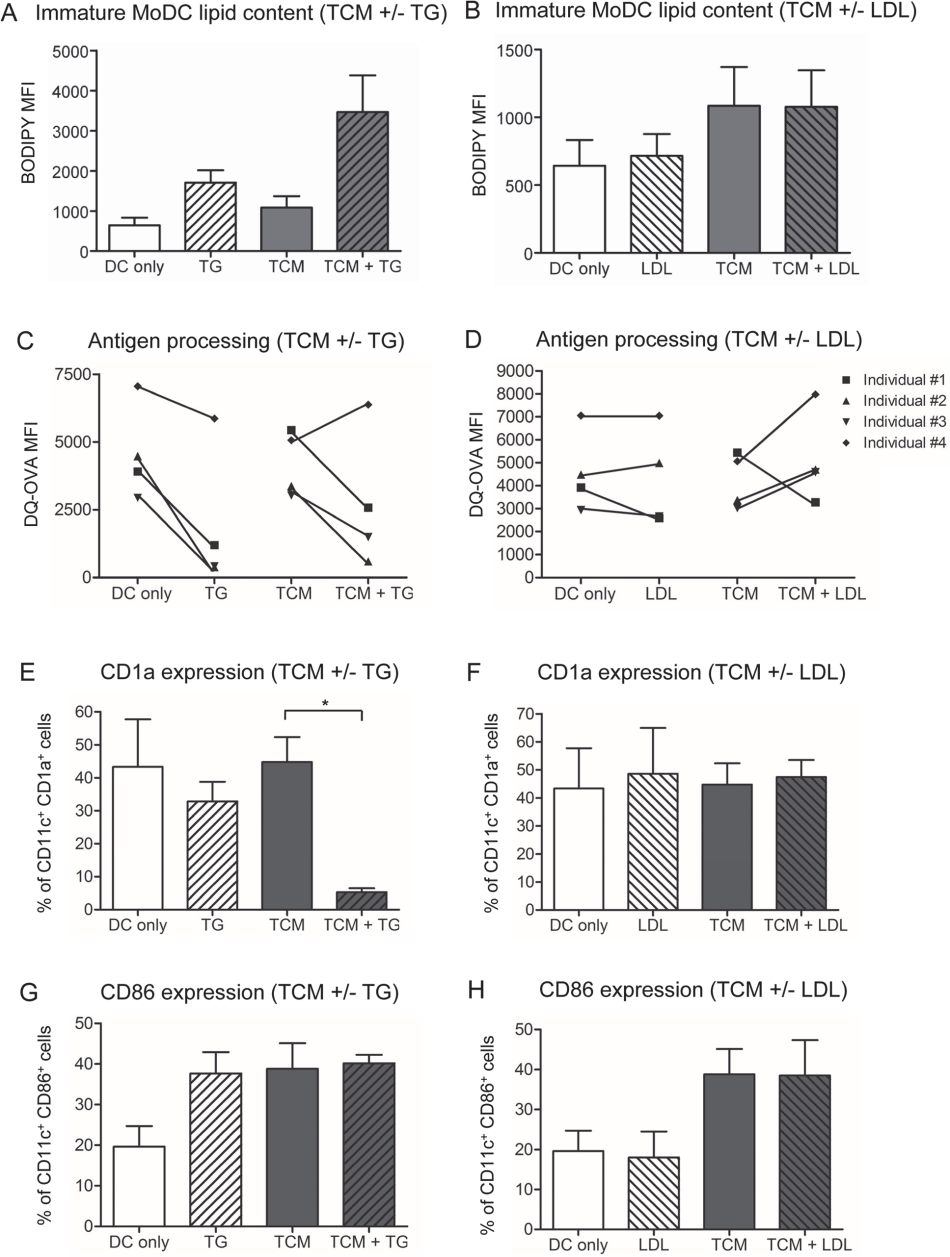
Triglyceride-rich lipoproteins and mesothelioma-derived soluble factors modulate immature MoDCs. During differentiation, immature MoDCs were exposed to 50% JU77 TCM, with the addition of either triglyceride-rich lipoproteins (TG) or low-density lipoproteins (LDL). The lipid content of MoDCs exposed to JU77 TCM +/- TG (A) or JU77 TCM +/- LDL (B) was measured using BODIPY MFI. The DQ-OVA assay was used to assess the antigen processing capacity of MoDCs exposed to JU77 TCM +/- TG (C) or JU77 TCM +/- LDL (D). Expression of CD1a and CD86 was also examined on MoDCs exposed to JU77 TCM +/- TG (E and G) or JU77 TCM +/- LDL (F and H). Data is from 4 individuals and is shown as mean ± SEM. * = p < 0.05.

Since other studies have shown that triglyceride-laden DCs in cancer patients are functionally disabled [12], we then investigated the combined influence of mesothelioma-derived factors and lipoproteins on DC lipid accumulation. To do this, MoDCs were differentiated in the presence of JU77 TCM and TG-rich lipoproteins or low-density lipoproteins (LDL; a cholesterol-rich lipoprotein). Addition of TG-rich lipoproteins, but not LDL, to TCM resulted in a greater increase in iMoDC lipid content compared to TCM only (Fig 5A and 5B). Furthermore, iMoDCs exposed to TG-rich lipoproteins (either alone or in combination with TCM), but not LDL, had reduced antigen processing capacity (Fig 5C and 5D). Changes to iMoDC surface phenotype were also examined. Addition of TG-rich lipoproteins, but not LDL, to TCM resulted in a significant decrease in the percent of iMoDCs expressing CD1a relative to DCs exposed to TCM only (Fig 5E and 5F). However, addition of either lipoprotein to TCM did not affect the percent of cells expressing CD86, relative to TCM only (Fig 5G and 5H). These results suggest that TG-rich lipoproteins, but not LDL, promote further increases in tumor-driven DC lipid accumulation, which is associated with impaired DC antigen processing ability and reduced CD1a expression.

Taken together, the data above suggests that mesothelioma cells and their secreted products promote iMoDC lipid accumulation. However, modulation of iMoDC function, phenotype and cytokine production appears to require close contact with tumor cells. A likely physiological site where these cell-to-cell events occur is the tumor microenvironment.

### Mesothelioma-derived factors alter mature MoDC cytokine production, but not lipid content

The data above shows that mesothelioma cells and their products modulate the DC differentiation process. The next series of experiments investigated the effects of mesothelioma-derived factors on MoDCs maturing in response to a bacterial stimulus, LPS. Immature MoDCs co-exposed to LPS and JU77 TCM (S5A Fig) did not demonstrate significant changes in lipid content (S5B Fig). Exposure to TCM during maturation did not alter the percent of cells expressing CD1a, HLA-DR, CD86 or CD80 (S5C–S5F Fig).

The primary function of mature DCs is to present processed antigen in MHC class I and class II molecules to T cells. If DCs have been appropriately activated then antigen-specific T cells will proliferate. The effect of tumor-derived factors on the ability of mature MoDCs to stimulate T cell proliferation was measured using the allogeneic MLR assay. Exposure to TCM did not affect the ability of mature MoDCs to stimulate CD4^+^ or CD8^+^ T cell proliferation (S5G and S5H Fig). These results suggest that tumor-driven alterations to MoDC lipid content, phenotype and function occur during MoDC differentiation, rather than maturation.

Mature MoDCs exposed to TCM produced lower levels of the pro-inflammatory cytokines IL-12p70 (a decrease from 448 ± 88.8 pg/ml to 52.6 ± 50.2 pg/ml) and TNF-α (a decrease from 2,265 ± 234.8 pg/ml to 1,930 ± 397.6 pg/ml) compared to mature MoDCs cultured without TCM (n = 2 healthy volunteers). Skewing DCs away from the production of pro-inflammatory cytokines may impair the generation of anti-mesothelioma T cell responses.

### Mesothelioma-derived factors may also influence lipid accumulation in immature murine bone marrow-derived DCs

**Fig 6:**
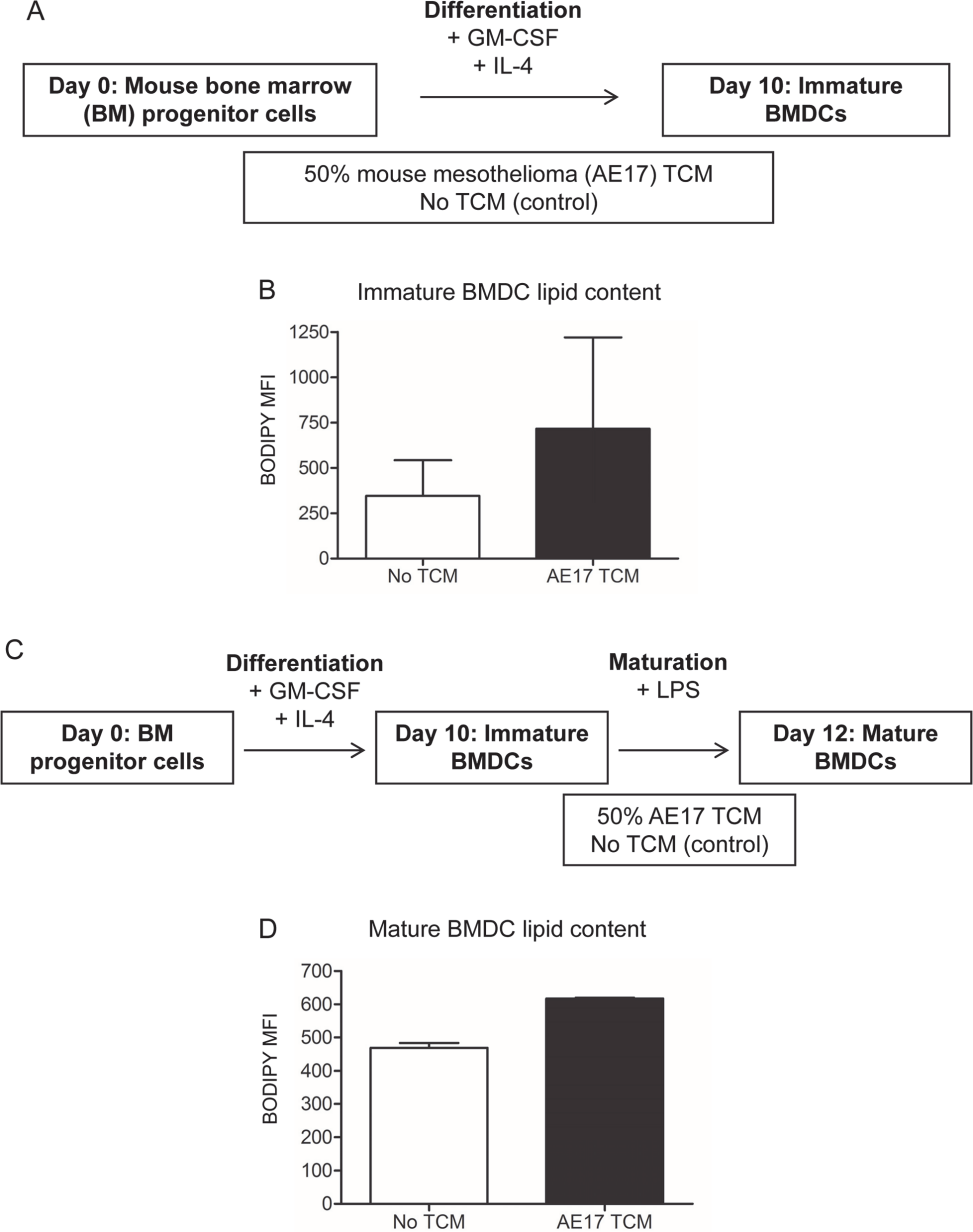
Mesothelioma-derived factors may promote lipid accumulation in immature bone marrow-derived murine DCs. Murine bone marrow (BM) progenitor cells cultured with GM-CSF and IL-4 were exposed to TCM from the AE17 mesothelioma cell line (A). At day 10, immature BMDC lipid levels were measured by BODIPY staining (B). Immature BMDCs were matured for 2 days using LPS and were co-exposed to 50% AE17 TCM (C) before lipid levels were analyzed (D). Pooled data in (B) and (D) is from 2 experiments. All data is shown as mean ± SEM.

Similar studies using murine bone marrow-derived DCs (BMDCs) were conducted to determine if factors derived from murine AE17 mesothelioma tumors could also influence DC lipid accumulation. Differentiating BMDCs exposed to AE17 TCM (Fig 6A) appeared to contain higher lipid levels than BMDCs cultured without TCM, but the differences did not reach statistical significance (Fig 6B). LPS-matured BMDCs exposed to AE17 TCM contained similar lipid levels relative to the controls (Fig 6C and 6D).

### Tumor-associated dendritic cells from mesothelioma-bearing mice contain high levels of lipid

**Fig 7:**
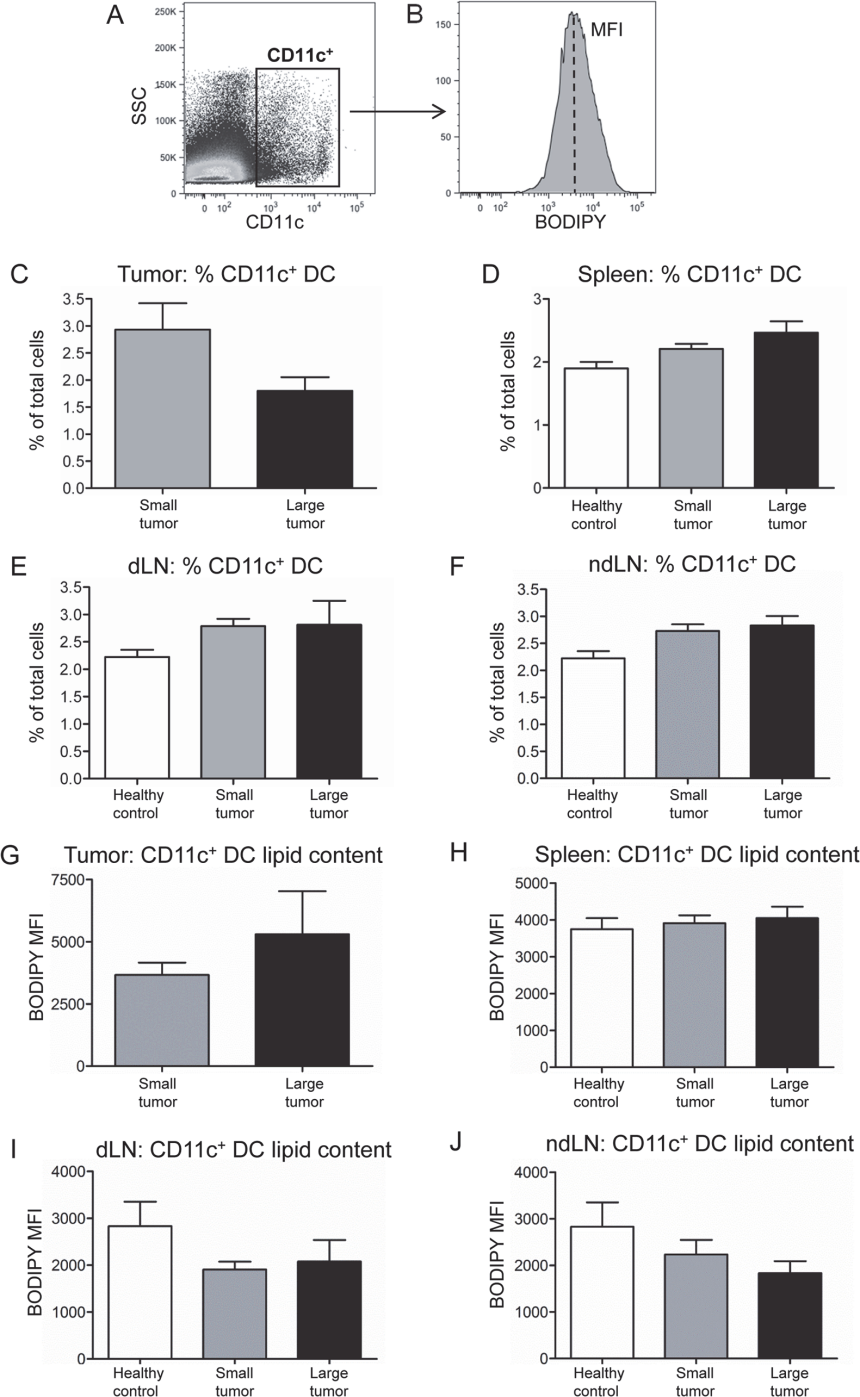
Tumor-DCs accumulate lipid and reduce numerically with disease progression. Mice were inoculated with 5 x 10^5^ AE17 mesothelioma cells and tumors allowed to develop into small (< 40 mm^2^) or large (> 80 mm^2^) tumors. Total CD11c^+^ DCs within tumors and lymphoid organs were identified (A) and lipid levels measured using BODIPY staining shown as MFIs of CD11c^+^ DCs; representative samples shown (B). Pooled data show the proportions of CD11c^+^ DCs within tumors (C), spleens (D), dLN (E) and ndLN (F). Pooled data show the lipid content of CD11c^+^ DCs in AE17 tumors (G), spleens (H), dLN (I) and ndLN (J). Lymphoid organs from tumor-bearing mice were compared to those from healthy mice: n = 18 mice with small tumors, n = 9 mice with large tumors and n = 8 healthy control mice. All pooled data are shown as mean ± SEM.

The next series of experiments investigated if similar results would be seen in vivo using the AE17 murine mesothelioma model. CD11c^+^ DC numbers and lipid levels were examined in mice bearing small (< 40 mm^2^) or large (> 80 mm^2^) mesothelioma tumors (Fig 7A and 7B). The proportion of tumor-infiltrating CD11c^+^ DCs decreased in large tumors compared to small tumors (Fig 7C). In contrast, CD11c^+^ DC proportions remained stable or were slightly elevated in the spleens, draining lymph nodes (dLN) and non-draining lymph nodes (ndLN) of tumor-bearing mice relative to healthy mice (Fig 7D–7F). CD11c^+^ DCs in large tumors appeared to contain higher lipid levels than DCs from small tumors (Fig 7G). The lipid content of CD11c^+^ DCs from spleens did not differ between tumor-bearing and healthy mice (Fig 7H). Unexpectedly, DCs in dLN and ndLN of tumor-bearing mice had lower lipid content than DCs in lymph nodes of healthy mice (Fig 7I and 7J). These data show that modulation of DC numbers and lipid content was restricted to the tumor microenvironment.

The lipid content of tumor and spleen tissue from tumor-bearing mice was also examined. Representative images of tumor and spleen morphology are shown in S6A and S6B Fig. Unstained (S6C and S6D Fig) and BODIPY-stained (S6E and S6F Fig) tissue sections were examined using fluorescence microscopy. Representative images show that tumor tissue contains neutral lipids in droplet-like structures (S6E Fig), whilst this is absent in spleen (S6F Fig). Lipid droplets in the tumor microenvironment may serve as a source of lipids for the observed lipid accumulation in tumor-associated DCs.

### Lipid levels in DC subsets are altered in mesothelioma-bearing mice

**Fig 8:**
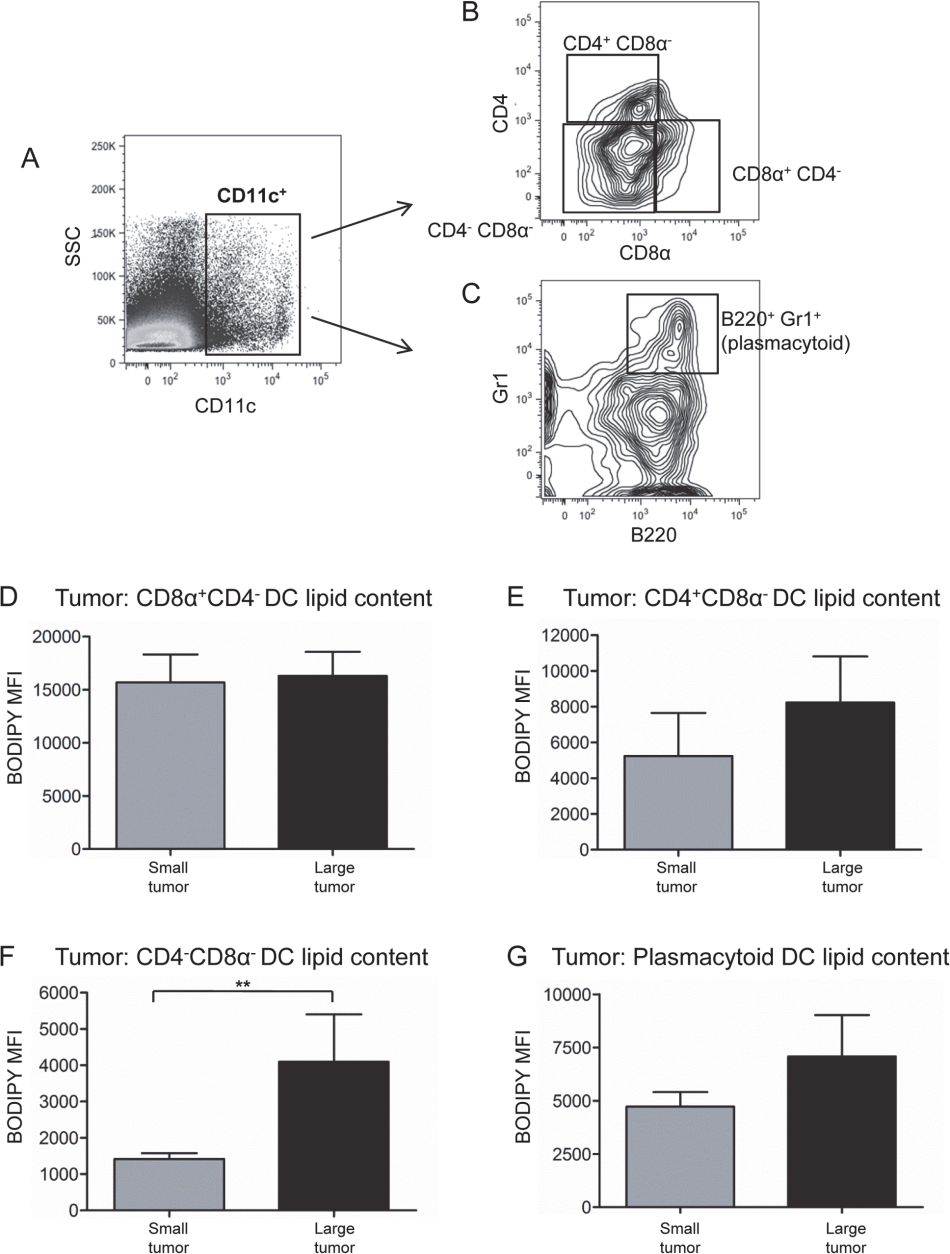
Lipid levels of three DC subsets increase in large mesothelioma tumors. DC subsets were identified by first gating on CD11c^+^ DCs (A). Expression of CD4 versus CD8α was used to identify CD4^+^CD8α ^-^, CD8α ^+^CD4^-^ and CD4^-^CD8α ^-^ DC subsets (B). Plasmacytoid DCs were identified as B220^+^Gr1^+^ cells (C). Lipid levels, shown as BODIPY MFI, were measured for CD8α ^+^CD4^-^ DCs (D), CD4^+^CD8α^-^ DCs (E), CD4^-^CD8α^-^ DCs (F) and plasmacytoid DCs (G) in small and large AE17 tumors: n = 18 mice with small tumors and n = 9 mice with large tumors. Pooled data are shown as mean ± SEM. ** = p < 0.005.

Dendritic cells represent a heterogeneous population of cells comprised of several different subsets, each with different but related functions [22]. Four DC subsets were examined (Fig 8A–8C). CD8α^+^CD4^-^ DCs play a key role in anti-tumor immunity due to their ability to cross-present tumor antigen to CD8^+^ T cells [23]. The CD8α^-^ subsets, i.e. CD4^+^CD8 α^-^ and CD4^-^CD8 α^-^ are described as conventional DCs that induce T helper cell responses [23]. CD11c^+^B220^+^Gr1^+^ plasmacytoid DCs play a key role in innate/anti-viral immunity [24].

The lipid levels of tumor-infiltrating CD8α^+^CD4^-^ DCs did not vary between small and large tumors (Fig 8D). In contrast, tumor-infiltrating CD4^+^CD8α^-^, CD4^-^CD8α^-^ and plasmacytoid DCs contained higher lipid levels in large tumors than small tumors (Fig 8E–8G). The lipid levels of splenic DC subsets did not change (S7A Fig) and DC subsets in dLN and ndLN of tumor-bearing mice demonstrated reduced lipid levels relative to healthy controls (S7B and S7C Fig).

**Fig 9:**
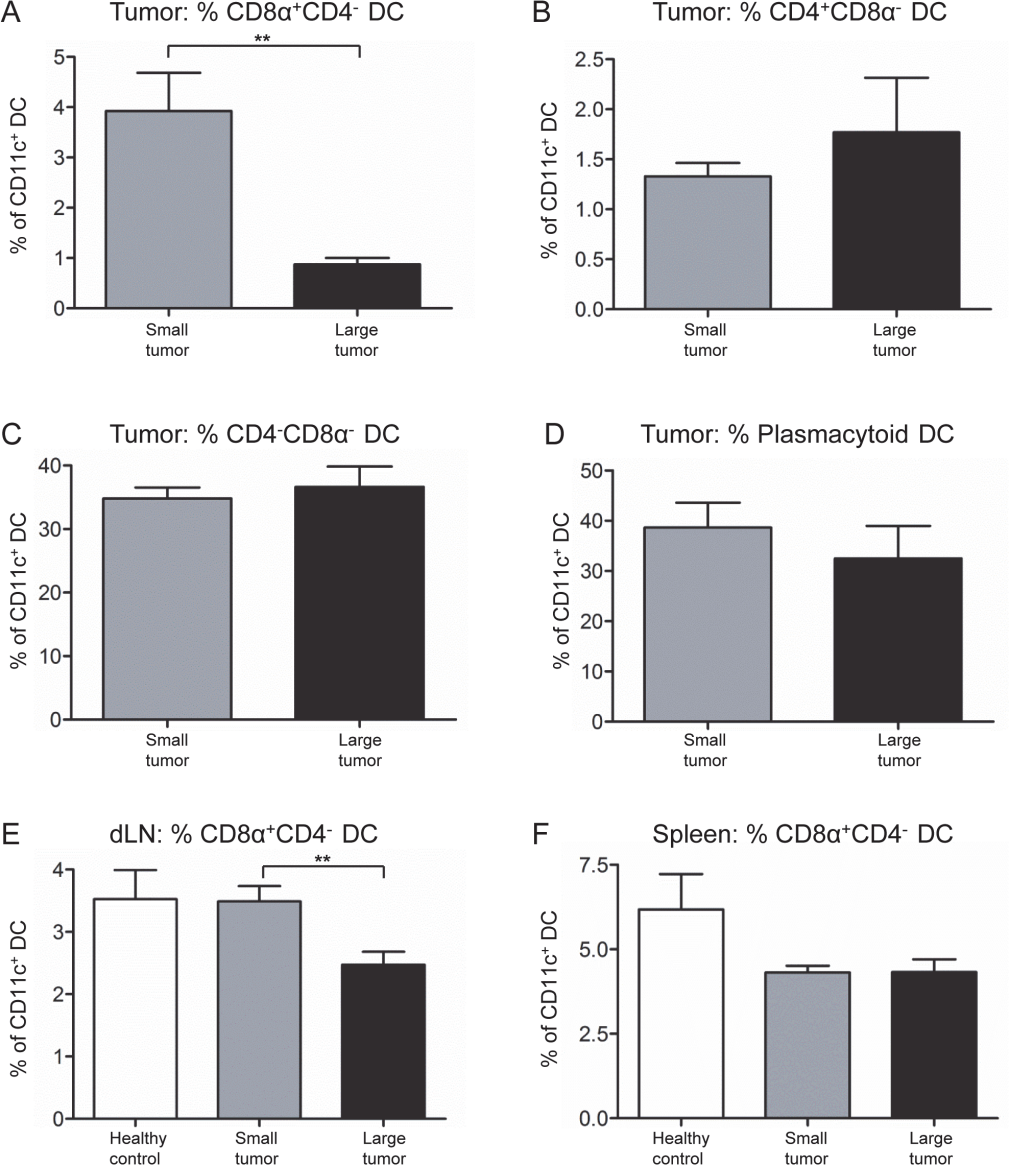
CD8α^+^CD4^-^ DC proportions decrease in mice with large mesothelioma tumors. DC subsets were identified as described in Fig 8. The proportions of CD8α^+^CD4^-^ DCs (A), CD4^+^CD8α^-^ DCs (B), CD4^-^CD8α^-^ DCs (C) and plasmacytoid DCs (D) within small versus large AE17 tumors were compared. The proportion of CD8α^+^CD4^-^ DCs in dLN (E) and spleens (F) of tumor-bearing mice were compared to healthy mice: n = 18 mice with small tumors, n = 9 mice with large tumors and n = 8 healthy control mice. Pooled data are shown as mean ± SEM. ** = p < 0.005.

The proportion of CD8α^+^CD4^-^ DCs decreased significantly in large tumors compared to small tumors (Fig 9A). The proportion of other DC subsets in tumors did not change (Fig 9B–9D). Similar reductions in the percent of CD8α^+^CD4^-^ DCs were seen in the dLN of large tumor-bearing mice (Fig 9E) and in the spleens of small and large tumor-bearing mice (Fig 9F). Other DC subsets in lymphoid organs of tumor-bearing mice did not change with tumor size (S8A–S8C Fig). These results suggest that increases in the lipid content of DC subsets occur mainly within the tumor. However, reductions in the proportions of CD8α^+^CD4^-^ DCs extend beyond the tumor.

### Reducing in vivo antigen presentation with increasing tumor burden

**Fig 10:**
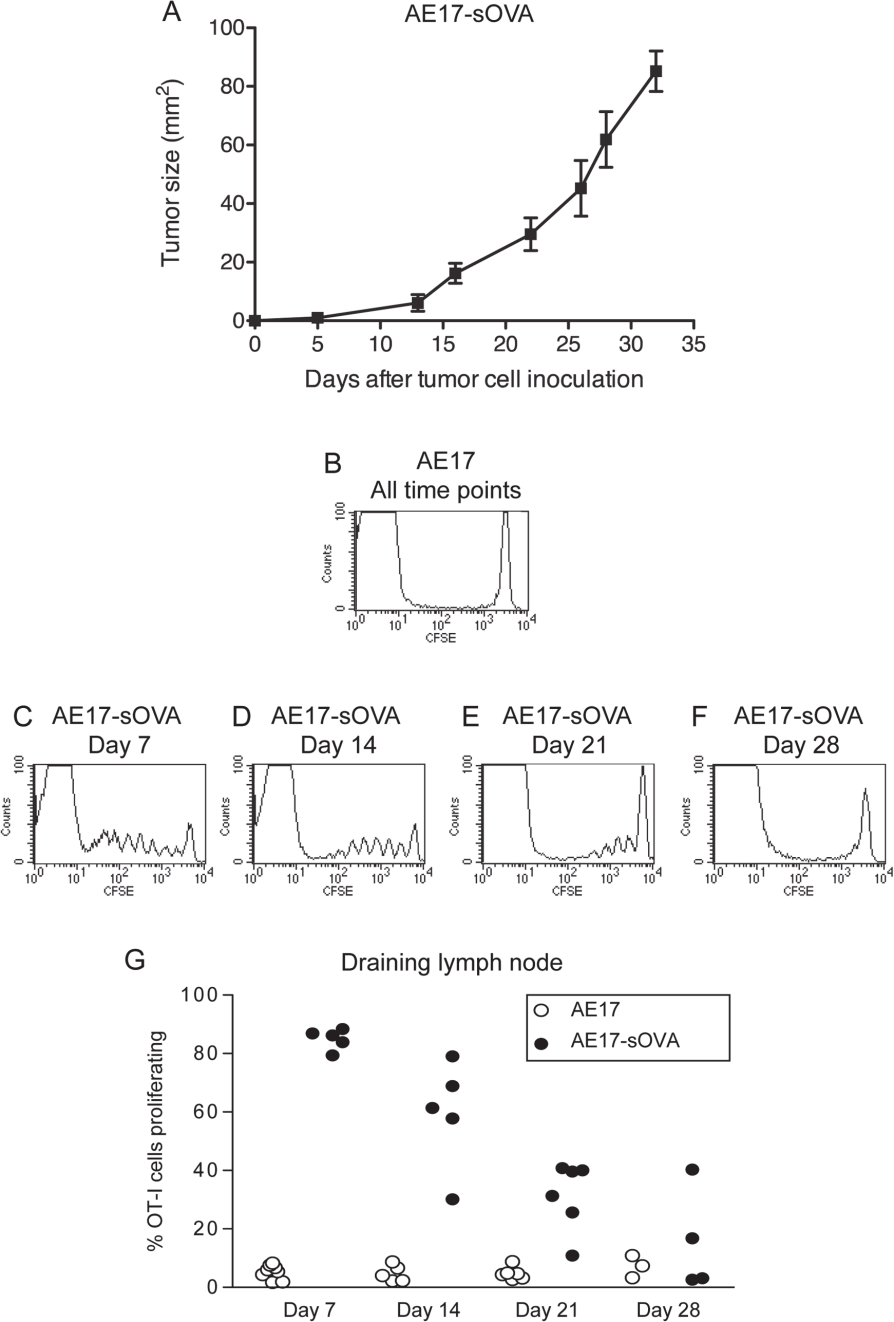
Tumor-antigen-specific CD8^+^ T cell proliferation in draining lymph nodes decreases with increasing tumor burden. C57BL/6J mice were inoculated with 5 x10^5^ AE17 tumor cells, which were used as negative controls (B) or with AE17sOVA tumor cells (growth rate shown in A; n = 10 mice; data shown as mean ± SEM) on day 0. CFSE-labelled, CD8^+^, class I restricted, OVA-specific T cells from OT-1 mice were adoptively transferred at days 4, 11, 18 and 25 into the tumor-bearing mice. The dLNs were harvested from recipient mice three days post transfer such that FACS analysis was on days 7 (C, G), 14 (D, G), 21 (E, G) and 28 (F, G) post tumor cell inoculation. The lymph nodes were prepared as single cell suspensions and stained for CD8 for re-isolation of CFSE-labelled OT-1 cells. FACS analysis was performed by gating on CD8^+^ T cells. Representative FACS profiles shown in B-F. Results from individual mice from 1 experiment with 3–6 mice/group (G).

The next experiments were designed to dissect out the in vivo relationship between tumor burden and tumor antigen presentation to CD8^+^ T cells in dLNs. A kinetic study was undertaken in which CFSE-labelled, CD8^+^ T cells prepared from the lymph nodes and spleens of OT-1 mice were adoptively transferred into recipient mice bearing either AE17 tumors (the negative control) or AE17sOVA tumors which express ovalbumin as a marker, or spy, tumor antigen and to which OT-1 T cells will respond by proliferating if the antigen is appropriately presented to them by local DCs [16]. The growth rate and tumor size of AE17sOVA is shown in Fig 10A. Throughout these experiments the proliferative responses of CFSE-labelled OT-1 cells in dLNs of recipient animals were analyzed 3 days post adoptive transfer. Responses were determined weekly over one month. A strong in vivo OT-1 proliferative response was seen in the dLNs of all mice bearing AE17sOVA tumors at days 7 and 14 (Fig 10C, 10D and 10G). This response started to diminish at day 21, disappearing by day 28 (Fig 10E, 10F and 10G). No OT-1 proliferation was seen in the dLNs at any time point in mice given control AE17 tumor cells (Fig 10B). These data show that decreasing tumor-antigen-specific CD8^+^ T cell proliferation in dLNs is associated with increasing tumor burden.

Taken together, the data suggests two mechanisms by which mesothelioma thwarts DC function: by promoting increases in DC lipid content and by reducing DC numbers.

### Attempts to normalize DC lipid levels and restore DC function

A previous study by Herber et al. [12] demonstrated that normalization of elevated DC lipid levels using an inhibitor of fatty acid synthesis, 5-tetradecycloxy-2-furoic acid (TOFA), restored the ability of DCs to stimulate allogeneic T cells. Thus, attempts were made to demonstrate a link between elevated DC lipid levels and altered DC function in our model. Human MoDCs were cultured in the presence of JU77 tumor cells (at a ratio of 1 DC: 10 JU77 cells) and 5 μg/ml TOFA or DMSO (the diluent control) was added for days 4–7 of culture. In the absence of tumor cells, TOFA and DMSO had no effect on DC lipid content, relative to the DC only control (S9A Fig). In the DC:tumor cell co-cultures, addition of TOFA did not; (i) reduce DC lipid levels (S9A Fig); (ii) restore DC antigen processing ability (S9B Fig); (iii) increase the ability of tumor-exposed DCs to induce allogeneic CD4^+^ and CD8^+^ T cell proliferation (S9C and S9D Fig). We also investigated the effects of TOFA on AE17 mesothelioma tumor growth in vivo. One day after tumor cell inoculation, mice were implanted with osmotic pumps containing TOFA or DMSO. From day 9 to day 13, both TOFA and DMSO appeared to slow tumor growth rate relative to untreated tumor-bearing controls (S9E Fig). However, after day 13, the tumor growth rate for all groups was similar. When tumors reached maximum size (100 mm^2^), the lipid content of DCs within spleens, dLNs and tumors was assessed using BODIPY staining and flow cytometry. In all organs examined, TOFA treatment did not lower DC lipid content relative to DCs from untreated tumor-bearing mice (S9F Fig). In summary, TOFA had little effect in lowering DC lipid content, in vitro or in vivo.

## Discussion

Recent studies have shown that DC lipid acquisition driven by tumor-derived factors contributes to DC dysfunction in several human cancers and in animal cancer models [12]. However, until now, DC lipid acquisition had not been investigated in mesothelioma. Thus, this study aimed to determine if mesothelioma tumor cells and/or their secreted products increased DC lipid content and modulated DC function. Our studies show that DC lipid acquisition occurred predominantly within the tumor microenvironment and was most likely driven via close proximity with mesothelioma tumor cells with evidence of a role for tumor-derived soluble factors. Two key DC-disabling associations were seen; lipid acquisition and reduced numbers of cross-presenting CD8α^+^CD4^-^ DCs. Increasing lipid levels and reducing numbers of CD8α^+^CD4^-^ DCs were associated with increasing tumor burden which was associated with decreasing T cell proliferative responses to tumor antigen presentation in dLNs.

Our in vitro studies showed that mesothelioma tumor cells and their secreted products exerted their effects on monocyte precursors differentiating into immature DCs. Stronger effects on iMoDC phenotype, antigen processing function and cytokine production were seen in co-cultures with tumor cells compared to tumor-derived factors in TCM. Thus, DC modulation required close proximity with tumor cells, a situation likely to occur in the tumor microenvironment. Furthermore, tumor-derived factors form a concentration gradient that would be highest within the tumor, leading to more powerful effects on DCs closely juxtaposed against tumor cells than at other sites [25]. The in vivo studies confirmed this hypothesis. These data suggest that DC precursors recruited from blood into the mesothelioma microenvironment interact with tumor cells, and their soluble products, which interfere with their differentiation into functional immature DCs.

We did not identify the mechanism leading to DC dysfunction, but tumor cell-derived factors including gangliosides [26], VEGF, IL-10 and TGF-β [2–5] may have a role. Examination of DC subsets gives further insights into possible mechanisms, and our studies showed that two mechanisms may disable DC function. Firstly, lipid accumulation by tumor-associated CD4^+^CD8α^-^, CD4^-^CD8α^-^ and plasmacytoid DCs implied that these subsets were impaired, however further functional studies such as antigen uptake and presentation assays and migration assays are required. This contrasts with a previous study, where tumor-derived factors promoted lipid acquisition by conventional, but not plasmacytoid, DCs [12]. This discrepancy may be due to differences in the markers used to identify DC subsets, or differences in tumor models and/or cancer types. The other mechanism observed was the proportional reduction of tumor-associated CD8α^+^CD4^-^ DCs as tumor size increased. Interestingly, this reduction was not associated with lipid accumulation. These observed modulations most likely occur during DC differentiation, as DC precursors differentiate into immature DCs at the tumor site [27].

The in vitro studies showed that mesothelioma cells and/or their factors initiated an early, but incomplete, maturation of iMoDCs, as evidenced by increased CD86 and reduced CD1a expression. Inappropriate maturation within the tumor may decrease DC capacity to take up and process antigen, leading to impaired generation of anti-tumor immunity. In other cancer models, ‘semi-mature’ DCs are unable to process antigen or stimulate T cell proliferation [28–30].

The changes in murine tumor-associated DCs and in human iMoDC function after exposure to mesothelioma cells were accompanied by increased intracellular lipid levels, with the in vitro iMoDCs showing decreased antigen processing ability. These observations are in agreement with the only other study addressing the functional ability of lipid-laden DCs in cancer [12], and imply that DCs in mesothelioma have limited capacity to take up and/or process tumor cells. Reduced processing of tumor-associated antigens may decrease antigen presentation in lymph nodes, inhibiting the generation of tumor-specific T cells [30], which may explain the observed reduction in tumor-antigen-specific CD8^+^ T cell proliferation in tumor-draining lymph nodes in our in vivo model. We attempted to establish a link between DC lipid accumulation and dysfunction by treating tumor-exposed MoDCs with a lipid-lowering agent, TOFA. However, TOFA treatment did not normalize DC lipid levels and restore DC function in our model, nor was it able to slow mesothelioma tumor growth. This may have been due to the confounding effects we observed with the TOFA diluent control, DMSO. DMSO is a common vehicle for several agents, however our results suggest that caution must be exercised when using agents solubilized in DMSO to treat DCs. In our model, further studies are required using different lipid-lowering agents, ideally those which have an aqueous diluent.

DC lipid accumulation was also associated with alterations in DC surface phenotype and cytokine production, which is indicative of altered DC function. Lipid-laden iMoDCs demonstrated reduced CD1a expression, which may decrease presentation of tumor-associated lipid antigens and reduce activation of lipid-specific T cells [31,32]. In cancer patients, reductions in tumor-infiltrating CD1a^+^ DCs are associated with poor prognosis [33,34]. The specific trigger for decreased CD1a expression in our studies is not known, but tumor-derived factors such as prostanoids [9] and gangliosides [26] are known to inhibit CD1a expression. In addition, lipid accumulation may lead to increased production of lipid mediators which activate transcription factors leading to downregulation of CD1a expression [35] and altered DC cytokine production [36]. Our results showed that tumor-exposed, lipid-laden iMoDCs increased production of the anti-inflammatory cytokine IL-10, suggesting skewing towards a tolerogenic profile. Thus, several mechanisms may modulate DC differentiation in the tumor microenvironment. Exactly how lipid accumulates is uncertain, but one possibility is that factors secreted by tumor cells may promote upregulation of scavenger receptors, such as scavenger receptor A, on DCs, leading to increased uptake of plasma lipids [12]. Other pathways, for example, involving lipoprotein lipase [37], may be involved. It is possible that tumor-derived factors increase DC production of lipoprotein lipase, leading to increased triglyceride hydrolysis and availability of lipids for subsequent uptake by DCs. Future studies will address these issues. Furthermore, we observed the presence of lipid-rich droplets in AE17 tumor tissue, which may act as a source of lipid for accumulation by tumor-associated DCs.

Whilst DC modulation predominantly occurred within mesothelioma tumors, some effects were seen in dLNs. DCs appeared to be able to migrate from tumors to dLNs. This was suggested by decreased tumor-infiltrating DCs with increasing mesothelioma tumor burden, and the trend towards increased DC numerical proportions in the dLN where we have previously shown they present antigen to T cells [16]. If immature DCs maintain their tolerogenic profile induced by tumor cells, as indicated by the increased IL-10 expression in our in vitro studies, this could lead to the induction of regulatory T cells [38], and/or T-helper (Th)-2 responses instead of Th-1 anti-tumor immune responses [9]. Mesothelioma-derived factors did not affect the ability of mature DCs to stimulate alloantigen-specific T cell proliferation, but did reduce mature DC production of pro-inflammatory cytokines, IL-12 and TNF-α, which may have downstream effects on the type of T cell response generated. As a result of reduced pro-inflammatory cytokine production, mature DCs may be unable to fully activate effector CD8^+^ T cells and Th-1 cells, or may promote tolerogenic T cells [39,40]. Furthermore, our in vivo mesothelioma studies showed that CD8α^+^CD4^-^ DCs were reduced in dLN and spleen, implying that cross-presentation of antigens is impaired in lymphoid organs [23]. Therefore, whilst DC modulation occurred primarily in the tumor, some mechanisms may be operating to disable DCs in lymphoid organs. This supported by our data that clearly shows decreasing tumor-antigen-specific CD8^+^ T cell proliferation is associated with increasing tumor burden. It is likely that the declining proportions of cross-presenting CD8α^+^CD4^-^ DCs and the presence of disabled and inappropriately activated lipid-laden tumor-associated DCs that migrated to dLNs account for this observation.

Interestingly, our data suggests that lipid accumulation may not be a main mechanism of DC modulation within lymph nodes. In contrast to the increase in lipid content of tumor-associated DCs, we observed that the lipid content of DCs within dLNs decreased with tumor progression. One possible explanation is that lipid-laden tumor-associated DCs are unable to migrate out of the tumor site and to dLNs. Reduced migration of DCs into dLNs may also explain the reduced stimulation of tumor-specific T cells we observed. However, since our data suggests that DCs may be able to migrate from tumors to dLNs, we therefore speculate that as DCs transit from the tumor site to the dLN, they metabolise their internal lipid stores to provide a source of energy for migration. Thus, DCs arriving at dLNs would have decreased lipid levels relative to DCs found in tumors, but would still be disabled and may be unable to stimulate tumor-specific T cells, as suggested by our in vivo data. Further studies are required to examine the functional capacity of DCs that have migrated from tumors to dLNs. Within lymph nodes, there are also resident DC subsets [23], and lipid accumulation may not occur in resident DCs due to the fact that they are distal from the tumor and as a result may be exposed to relatively low concentrations of tumor-derived factors. We also observed that mature MoDCs exposed to tumor-derived factors in TCM do not accumulate lipid, and this supports our speculation that tumor-induced modulation of DC lipid content occurs predominantly during DC differentiation in the tumor microenvironment and requires close proximity between DCs and tumor cells and/or relatively high concentrations of tumor-derived factors, such as those found in the tumor microenvironment. We hypothesize that immature DCs recruited into the tumor microenvironment could be particularly sensitive to changes to expression of genes and/or receptors involved in lipid metabolism and uptake [37], whilst mature DCs may be more resistant possibly because they have already responded to other maturation signals [28,41]. Thus, lipid accumulation appears to be a mechanism of modulating DCs within tumors, but not lymph nodes.

This study identified several strategies by which mesothelioma may impair DC function, and which could be clinically relevant. Reductions in murine tumor-infiltrating DCs occurred during disease progression, similar to human mesothelioma [42]. Other studies have shown reduced DC numbers in peripheral blood of cancer patients [2,29,43], which may alter anti-tumor immunity and affect prognosis [44]. Additionally, lipid accumulation, in particular, increased intracellular triglycerides, led to DC dysfunction in human and murine cancers [12]. Similarly, here we show that triglyceride-rich lipoproteins enhanced mesothelioma-driven DC lipid accumulation and that the mesothelioma microenvironment contains structures rich in neutral lipids. Further studies are required to investigate the mechanism of DC lipid accumulation, including the roles of scavenger receptor A and lipoprotein lipase. Furthermore, the mesothelioma microenvironment contains suppressive cytokines including VEGF and TGF-β [42]. JU77 mesothelioma cells secreted VEGF, which impairs DC maturation [3,4] and TGF-β, which inhibits DC maturation and promotes a tolerogenic phenotype [5,45]. Identification of other factors secreted by mesothelioma cells, and studies to determine which specific factors promote DC lipid accumulation, are required.

The mechanisms by which mesothelioma modulates DCs represent potential therapeutic targets. This study suggests that tumor-infiltrating and dLN DC function has to be rescued. Immunotherapy offers several options, for example, targeting intra-tumoral DCs in situ with cytokines or pathogenic stimuli, leading to DC activation and effective anti-tumor responses [46,47]. Blocking mesothelioma-derived factors and/or their signalling pathways with neutralizing antibodies is another approach [48,49]. It has also been shown that intracellular inhibitors of fatty acid synthesis can be used to reduce DC lipid levels and restore DC function in murine cancer models [12]. However, in our model, this approach was unsuccessful, which suggests that we may need to test different lipid-lowering agents and/or target different pathways. For example, lowering plasma triglyceride-rich lipoproteins or blocking DC lipid uptake via scavenger receptors may be beneficial. As lipid accumulation may be one of several mechanisms that tumors use to disable DCs, lipid-lowering therapies combined with immunotherapy may produce more potent anti-tumor effects. This was demonstrated by Herber et al. [12], who showed that combining TOFA with anti-tumor vaccines slowed tumor growth, whilst the monotherapies had little effect. Similarly our study showing that TOFA treatment did not slow murine mesothelioma tumor growth also supports the idea that combination lipid-lowering/immunotherapy may be more effective.

In summary, this study showed that mesothelioma tumors and their secreted factors promote DC lipid accumulation, reduce DC numbers, in particular cross-presenting CD8α^+^CD4^-^ DCs, impair antigen processing and antigen presentation ability and skew DCs to produce tolerogenic cytokines. It is likely that these mechanisms are operating within the mesothelioma microenvironment and sabotage the ability of DCs to generate anti-mesothelioma immune responses, in particular the ability to stimulate tumor-specific CD8^+^ T cell proliferation in dLNs. Lipid-lowering therapies combined with another therapy, such as immunotherapy, may restore DC function, leading to the successful generation of anti-tumor immunity and an improved patient outcome.

## Supporting Information

S1 Fig JU77 lipid content does not change during co-culture with MoDCs. Immature MoDCs were co-cultured with varying ratios of JU77 cells as described in Fig 1A, and then analyzed using flow cytometry for CD11c expression and lipid content. JU77 cells were identified as CD11c negative cells (CD11c^neg^ gate; A) and JU77 lipid content was measured using the MFI of BODIPY staining (B). The lipid content of JU77 cells at various DC:JU77 tumor cell ratios is shown (C). Data is from 1 experiment for JU77 only and 7 experiments for DC:JU77 ratios of 10:1, 1:1 and 1:10.
(TIF)

S2 Fig Met5A cells do not affect iMoDC antigen processing or surface marker expression. During differentiation, MoDCs were co-cultured with JU77 tumor cells or a control cell line, Met5A, at a ratio of 1 DC:10 JU77 or Met5A cells. Following co-culture, iMoDC antigen processing ability was assessed using the DQ-OVA assay (A). The percent of iMoDCs positive for CD1a (B), CD86 (C) and CD80 (D) were examined. iMoDC expression levels of CD86 (E) and CD80 (F) were also measured using the MFIs of CD86 and CD80 staining. Data is from 3 individuals and is shown as mean ± SEM. * = p < 0.05.
(TIF)

S3 Fig Mesothelioma cells and soluble factors do not alter the ability of iMoDCs to stimulate T cells. Immature MoDCs were co-cultured with varying ratios of JU77 cells during DC differentiation, as described in Fig 1A. Following co-culture, the ability of immature MoDCs to stimulate CD4^+^ (A) and CD8^+^ (B) T cell proliferation was assessed using the allogeneic MLR. Immature MoDCs exposed to varying concentrations of JU77 TCM during differentiation (described in Fig 4A) were also assessed for their ability to stimulate CD4^+^ (C) and CD8^+^ (D) T cell proliferation. Pooled data is from 3 individuals and shown as mean ± SEM.
(TIF)

S4 Fig Mesothelioma tumor-derived factors do not affect iMoDC HLA-DR and CD80 expression. HLA-DR and CD80 expression were measured on iMoDCs cultured in the presence of varying concentrations of JU77 TCM. Pooled data of the percent of iMoDCs expressing HLA-DR (A) and CD80 (C) and surface expression levels (shown as MFIs) of HLA-DR (B) and CD80 (D) on iMoDCs is from 6 individuals and shown as mean ± SEM.
(TIF)

S5 Fig Mesothelioma tumor-derived factors do not affect mature DCs. Immature human MoDCs were matured for 2 days using LPS with or without 50% JU77 TCM (A). Lipid levels (shown as MFI; B), and expression of CD1a (C), HLA-DR (D), CD86 (E) and CD80 (F) were measured using flow cytometry. The ability of mature MoDCs to stimulate T cell proliferation was measured using the MLR assay involving CFSE-labelled allogeneic T cells. Mature DCs were co-cultured with varying ratios of T cells, at day 8 non-adherent cells were stained to identify CD4^+^ and CD8^+^ T cells. The percent of T cell proliferation was calculated based on the loss of CFSE staining intensity of the parent peak. The ability of mature MoDCs cultured with or without JU77 TCM to stimulate CD4^+^ (G) and CD8^+^ (H) T cell proliferation is shown. Pooled data from 4 individuals is shown as mean ± SEM.
(TIF)

S6 Fig Tumor tissue, but not spleen, contains neutral lipids. Tumor and spleen sections from AE17 tumor-bearing mice were stained with haematoxylin and eosin (H&E) for general morphology (A and B); scale bars = 200 μm. Unstained sections (C and D) were used as controls for BODIPY-stained tumor and spleen sections (E and F) visualised using fluorescence microscopy; scale bars = 100 μm. Representative images from one experiment are shown.
(TIF)

S7 Fig Lipid content of DC subsets is reduced in LNs and does not change in spleens of tumor-bearing mice. Lipid levels (shown as BODIPY MFIs) of DC subsets were measured in spleens (A), dLNs (B) and ndLNs (C) of tumor-bearing and healthy control mice: n = 18 mice with small tumors, n = 9 mice with large tumors and n = 8 healthy control mice. Pooled data are shown as mean ± SEM.
(TIF)

S8 Fig Proportions of CD4^+^CD8α^-^ DCs, CD4^-^CD8α^-^ DCs and plasmacytoid DCs in lymphoid organs do not change with tumor size. The proportions of DC subsets within spleens (A), dLNs (B) and ndLNs (C) of tumor-bearing and healthy mice were compared: n = 18 mice with small tumors, n = 9 mice with large tumors and n = 8 healthy control mice. Pooled data are shown as mean ± SEM. ** = p < 0.005.
(TIF)

S9 Fig TOFA treatment is unable to normalize DC lipid levels. MoDCs were cultured alone or in the presence of JU77 tumor cells (1 DC: 10 JU77 cells). 5 μg/ml TOFA or DMSO was added to MoDC cultures for days 4–7. On day 7, MoDC lipid content (A) and antigen processing capacity (B) were assessed. The ability of MoDCs to stimulate CD4+ T cell (C) and CD8+ T cell (D) proliferation was measured using an MLR assay. Mice inoculated with AE17 tumor cells were left untreated (AE17 only) or implanted with osmotic pumps containing TOFA or DMSO on day 1 following tumor inoculation (black arrow; E). Mice received a dose of 1 μg TOFA/hour for 14 days; the treatment period is indicated by the shaded bar in (E). Tumor size (in mm2) was measured daily (E). DC lipid content in spleens, dLNs and tumors of untreated, TOFA- or DMSO-treated tumor-bearing mice was assessed (F). All data are shown as mean ± SEM. Data in (A)–(D) are pooled from 3 individuals. For murine studies shown in (E) and (F), n = 5 mice/group. Representative data are shown in (F). * = p < 0.05; ** = p < 0.005.
(TIF)

## References

[1] Gabrilovich D. Mechanisms and functional significance of tumour-induced dendritic-cell defects. Nat Rev Immunol. 2004; 4: 941–952. 15573129 10.1038/nri1498

[2] Almand B, Resser JR, Lindman B, Nadaf S, Clark JI, Kwon ED, et al Clinical significance of defective dendritic cell differentiation in cancer. Clin Cancer Res. 2000; 6: 1755–1766. 10815894

[3] Gabrilovich DI, Chen HL, Girgis KR, Cunningham HT, Meny GM, Nadaf S, et al Production of vascular endothelial growth factor by human tumors inhibits the functional maturation of dendritic cells. Nat Med. 1996; 2: 1096–1103. 8837607

[4] Oyama T, Ran S, Ishida T, Nadaf S, Kerr L, Carbone DP, et al Vascular endothelial growth factor affects dendritic cell maturation through the inhibition of nuclear factor-KB activation in hemopoietic progenitor cells. J Immunol. 1998; 160: 1224–1232. 9570538

[5] Brown RD, Pope B, Murray A, Esdale W, Sze DM, Gibson J, et al Dendritic cells from patients with myeloma are numerically normal but functionally defective as they fail to up-regulate CD80 (B7-1) expression after huCD40LT stimulation because of inhibition by transforming growth factor-beta-1 and interleukin-10. Blood. 2001; 98: 2992–2998. 11698282 10.1182/blood.v98.10.2992

[6] Diao J, Zhao J, Winter E, Cattral MS. Tumors suppress in situ proliferation of cytotoxic T cells by promoting differentiation of Gr-1+ conventional dendritic cells through IL-6. J Immunol. 2011; 186: 5058–5067. 10.4049/jimmunol.1004125 21430223

[7] Yang AS, Lattime EC. Tumor-induced interleukin 10 suppresses the ability of splenic dendritic cells to stimulate CD4 and CD8 T-cell responses. Cancer Res. 2003; 63: 2150–2157. 12727833

[8] Allavena P, Piemonti L, Longoni D, Bernasconi S, Stoppacciaro A, Ruco L, et al IL-10 prevents the differentiation of monocytes to dendritic cells but promotes their maturation to macrophages. Eur J Immunol. 1998; 28: 359–369. 9485215 10.1002/(SICI)1521-4141(199801)28:01<359::AID-IMMU359>3.0.CO;2-4

[9] Sombroek CC, Stam AGM, Masterson AJ, Lougheed SM, Schakel MJAG, Meijer C, et al Prostanoids play a major role in the primary tumor-induced inhibition of dendritic cell differentiation. J Immunol. 2002; 168: 4333–4343. 11970975 10.4049/jimmunol.168.9.4333

[10] Caldwell S, Heitger A, Shen W, Liu Y, Taylor B, Ladisch S. Mechanisms of ganglioside inhibition of APC function. J Immunol. 2003; 171: 1676–1683. 12902465 10.4049/jimmunol.171.4.1676 PMC2849639

[11] Villablanca EJ, Raccosta L, Zhou D, Fontana R, Maggioni D, Negro A, et al Tumor-mediated liver X receptor-alpha activation inhibits CC chemokine receptor-7 expression on dendritic cells and dampens antitumor responses. Nat Med. 2010; 16: 98–105. 10.1038/nm.2074 20037595

[12] Herber DL, Cao W, Nefedova Y, Novitskiy SV, Nagaraj S, Tyurin VA, et al Lipid accumulation and dendritic cell dysfunction in cancer. Nat Med. 2010; 16: 880–886. 10.1038/nm.2172 20622859 PMC2917488

[13] Robinson BWS, Musk AW, Lake RA. Malignant mesothelioma. Lancet. 2005; 366: 397–408. 16054941 10.1016/S0140-6736(05)67025-0

[14] Manning LS, Whitaker D, Murch AR, Garlepp MJ, Davis MR, Musk AW, et al Establishment and characterization of five human malignant mesothelioma cell lines derived from pleural effusions. Int J Cancer. 1991; 47: 285–290. 1703129 10.1002/ijc.2910470219

[15] Ke Y, Reddel RR, Gerwin BI, Reddel HK, Somers AN, McMenamin MG, et al Establishment of a human in vitro mesothelial cell model system for investigating mechanisms of asbestos-induced mesothelioma. Am J Pathol. 1989; 134: 979–991. 2541616 PMC1879894

[16] Jackaman C, Bundell CS, Kinnear BF, Smith AM, Filion P, van Hagen D, et al IL-2 intratumoral immunotherapy enhances CD8+ T cells that mediate destruction of tumor cells and tumor-associated vasculature: A novel mechanism for IL-2. J Immunol. 2003; 171: 5051–5063. 14607902 10.4049/jimmunol.171.10.5051

[17] Sixt M, Kanazawa N, Selg M, Samson T, Roos G, Reinhardt DP, et al The conduit system transports soluble antigens from the afferent lymph to resident dendritic cells in the T cell area of the lymph node. Immunity. 2005; 22: 19–29. 15664156 10.1016/j.immuni.2004.11.013

[18] Parish CR. Fluorescent dyes for lymphocyte migration and proliferation studies. Immunol Cell Biol. 1999; 77: 499–508. 10571670 10.1046/j.1440-1711.1999.00877.x

[19] Mamotte CDS, Sturm M, Foo JI, van Bockxmeer FM, Taylor RR. Familial defective apolipoprotein B-100 (FDB): Effect of simvastatin therapy on LDL-receptor binding. Atherosclerosis. 1996; 125: 103–110. 8831932 10.1016/0021-9150(96)05867-4

[20] Lutz MB, Kukutsch N, Ogilvie ALJ, Robner S, Koch F, Romani N, et al An advanced culture method for generating large quantities of highly pure dendritic cells from mouse bone marrow. J Immunol Methods. 1999; 223: 77–92. 10037236 10.1016/s0022-1759(98)00204-x

[21] Cao X, Sugita M, van der Wel N, Lai J, Rogers RA, Peters PJ, et al CD1 molecules efficiently present antigen in immature dendritic cells and traffic independently of MHC class II during dendritic cell maturation. J Immunol. 2002; 169: 4770–4777. 12391186 10.4049/jimmunol.169.9.4770

[22] Shortman K, Liu YJ. Mouse and human dendritic cell subtypes. Nat Rev Immunol. 2002; 2: 151–161. 11913066 10.1038/nri746

[23] Pooley JL, Heath WR, Shortman K. Intravenous soluble antigen is presented to CD4 T cells by CD8- dendritic cells, but cross-presented to CD8 T cells by CD8+ dendritic cells. J Immunol. 2001; 166: 5327–5330. 11313367 10.4049/jimmunol.166.9.5327

[24] Nakano H, Yanagita M, Gunn MD. CD11c+B220+Gr-1+ cells in mouse lymph nodes and spleen display characteristics of plasmacytoid dendritic cells. J Exp Med. 2001; 194: 1171–1178. 11602645 10.1084/jem.194.8.1171 PMC2193516

[25] Gottfried E, Kreutz M, Mackensen A. Tumor-induced modulation of dendritic cell function. Cytokine Growth Factor Rev. 2008; 19: 65–77. 18061513 10.1016/j.cytogfr.2007.10.008

[26] Peguet-Navarro J, Sportouch M, Popa I, Berthier O, Schmitt D, Portoukalian J. Gangliosides from human melanoma tumors impair dendritic cell differentiation from monocytes and induce their apoptosis. J Immunol. 2003; 170: 3488–3494. 12646609 10.4049/jimmunol.170.7.3488

[27] Banchereau J, Briere F, Caux C, Davoust J, Lebecque S, Liu YJ, et al Immunobiology of dendritic cells. Annual Rev Immunol. 2000; 18: 767–811. 10837075 10.1146/annurev.immunol.18.1.767

[28] Kiertscher SM, Luo J, Dubinett SM, Roth MD. Tumors promote altered maturation and early apoptosis of monocyte-derived dendritic cells. J Immunol. 2000; 164: 1269–1276. 10640740 10.4049/jimmunol.164.3.1269

[29] Della Bella S, Gennaro M, Vaccari M, Ferraris C, Nicola S, Riva A, et al Altered maturation of peripheral blood dendritic cells in patients with breast cancer. Br J Cancer. 2003; 89: 1463–1472. 14562018 10.1038/sj.bjc.6601243 PMC2394334

[30] Gerner MY, Mescher MF. Antigen processing and MHC-II presentation by dermal and tumor-infiltrating dendritic cells. J Immunol. 2009; 182: 2726–2737. 10.4049/jimmunol.0803479 19234167 PMC2712950

[31] Coventry B, Heinzel S. CD1a in human cancers: A new role for an old molecule. Trends Immunol. 2004; 25: 242–248. 15099564 10.1016/j.it.2004.03.002

[32] Gerlini G, Tun-Kyi A, Dudli C, Burg G, Pimpinelli N, Nestle FO. Metastatic melanoma secreted IL-10 down-regulates CD1 molecules on dendritic cells in metastatic tumor lesions. Am J Pathol. 2004; 165: 1853–1863. 15579430 10.1016/S0002-9440(10)63238-5 PMC1618726

[33] Coventry BJ, Morton J. CD1a-positive infiltrating-dendritic cell density and 5-year survival from human breast cancer. Br J Cancer. 2003; 89: 533–538. 12888826 10.1038/sj.bjc.6601114 PMC2394362

[34] Goldman SA, Baker E, Weyant RJ, Clarke MR, Myers JN, Lotze MT. Peritumoral CD1a-positive dendritic cells are associated with improved survival in patients with tongue carcinoma. Arch Otolaryngol Head Neck Surg. 1998; 124: 641–646. 9639473 10.1001/archotol.124.6.641

[35] Gogolak P, Rethi B, Szatmari I, Lanyi A, Dezso B, Nagy L, et al Differentiation of CD1a- and CD1a+ monocyte-derived dendritic cells is biased by lipid environment and PPAR-gamma. Blood. 2007; 109: 643–652. 16968896 10.1182/blood-2006-04-016840

[36] Zeyda M, Saemann MD, Stuhlmeier KM, Mascher DG, Nowotny PN, Zlabinger GJ, et al Polyunsaturated fatty acids block dendritic cell activation and function independently of NF-KB activation. J Biol Chem. 2005; 280: 14293–14301. 15684433 10.1074/jbc.M410000200

[37] Le Naour F, Hohenkirk L, Grolleau A, Misek DE, Lescure P, Geiger JD, et al Profiling changes in gene expression during differentiation and maturation of monocyte-derived dendritic cells using both oligonucleotide microarrays and proteomics. J Biol Chem. 2001; 276: 17920–17931. 11279020 10.1074/jbc.M100156200

[38] Torres-Aguilar H, Aguilar-Ruiz SR, Gonzalez-Perez G, Munguia R, Bajana S, Meraz-Rios MA, et al Tolerogenic dendritic cells generated with different immunosuppressive cytokines induce antigen-specific anergy and regulatory properties in memory CD4+ T cells. J Immunol. 2010; 184: 1765–1775. 10.4049/jimmunol.0902133 20083662

[39] Moretto MM, Lawlor EM, Khan IA. Lack of interleukin-12 in p40-deficient mice leads to poor CD8+ T-cell immunity against Encephalitozoon cuniculi infection. Infect Immun. 2010; 78: 2505–2511. 10.1128/IAI.00753-09 20308292 PMC2876566

[40] Hilkens CMU, Kalinski P, de Boer M, Kapsenberg ML. Human dendritic cells require exogenous interleukin-12-inducing factors to direct the development of naive T-helper cells toward the Th1 phenotype. Blood. 1997; 90: 1920–1926. 9292525

[41] Granucci F, Vizzardelli C, Virzi E, Rescigno M, Ricciardi-Castagnoli P. Transcriptional reprogramming of dendritic cells by differentiation stimuli. Eur J Immunol. 2001; 31: 2539–2546. 11536151 10.1002/1521-4141(200109)31:9<2539::aid-immu2539>3.0.co;2-9

[42] Hegmans JPJJ, Hemmes A, Hammad H, Boon L, Hoogsteden HC, Lambrecht BN. Mesothelioma environment comprises cytokines and T-regulatory cells that suppress immune responses. Eur Respir J. 2006; 27: 1086–1095. 16540497 10.1183/09031936.06.00135305

[43] Beckebaum S, Zhang X, Chen X, Yu Z, Frilling A, Dworacki G, et al Increased levels of interleukin-10 in serum from patients with hepatocellular carcinoma correlate with profound numerical deficiencies and immature phenotype of circulating dendritic cell subsets. Clin Cancer Res. 2004; 10: 7260–7269. 15534100 10.1158/1078-0432.CCR-04-0872

[44] Inoshima N, Nakanishi Y, Minami T, Izumi M, Takayama K, Yoshino I, et al The influence of dendritic cell infiltration and vascular endothelial growth factor expression on the prognosis of non-small cell lung cancer. Clin Cancer Res. 2002; 8: 3480–3486. 12429638

[45] Bellone G, Carbone A, Smirne C, Scirelli T, Buffolino A, Novarino A, et al Cooperative induction of a tolerogenic dendritic cell phenotype by cytokines secreted by pancreatic carcinoma cells. J Immunol. 2006; 177: 3448–3460. 16920987 10.4049/jimmunol.177.5.3448

[46] Saito T, Takayama T, Osaki T, Nagai S, Suzuki T, Sato M, et al Combined mobilization and stimulation of tumor-infiltrating dendritic cells and natural killer cells with Flt3 ligand and IL-18 in vivo induces systemic antitumor immunity. Cancer Sci. 2008; 99: 2028–2036. 10.1111/j.1349-7006.2008.00907.x 19016763 PMC11158939

[47] Furumoto K, Soares L, Engleman EG, Merad M. Induction of potent antitumor immunity by in situ targeting of intratumoral DCs. J Clin Invest. 2004; 113: 774–783. 14991076 10.1172/JCI19762 PMC351319

[48] Biswas S, Guix M, Rinehart C, Dugger TC, Chytil A, Moses HL, et al Inhibition of TGF-beta with neutralizing antibodies prevents radiation-induced acceleration of metastatic cancer progression. J Clin Invest. 2007; 117: 1305–1313. 17415413 10.1172/JCI30740 PMC1838926

[49] Gabrilovich DI, Ishida T, Nadaf S, Ohm JE, Carbone DP. Antibodies to vascular endothelial growth factor enhance the efficacy of cancer immunotherapy by improving endogenous dendritic cell function. Clin Cancer Res. 1999; 5: 2963–2970. 10537366

