# Supplementary figures and images for: Mesothelioma Tumor Cells Modulate Dendritic Cell Lipid Content, Phenotype and Function
Source: PLoS One. 2015 Apr 17;10(4):e0123563. doi: 10.1371/journal.pone.0123563 (PMC4401725; doi:10.1371/journal.pone.0123563)

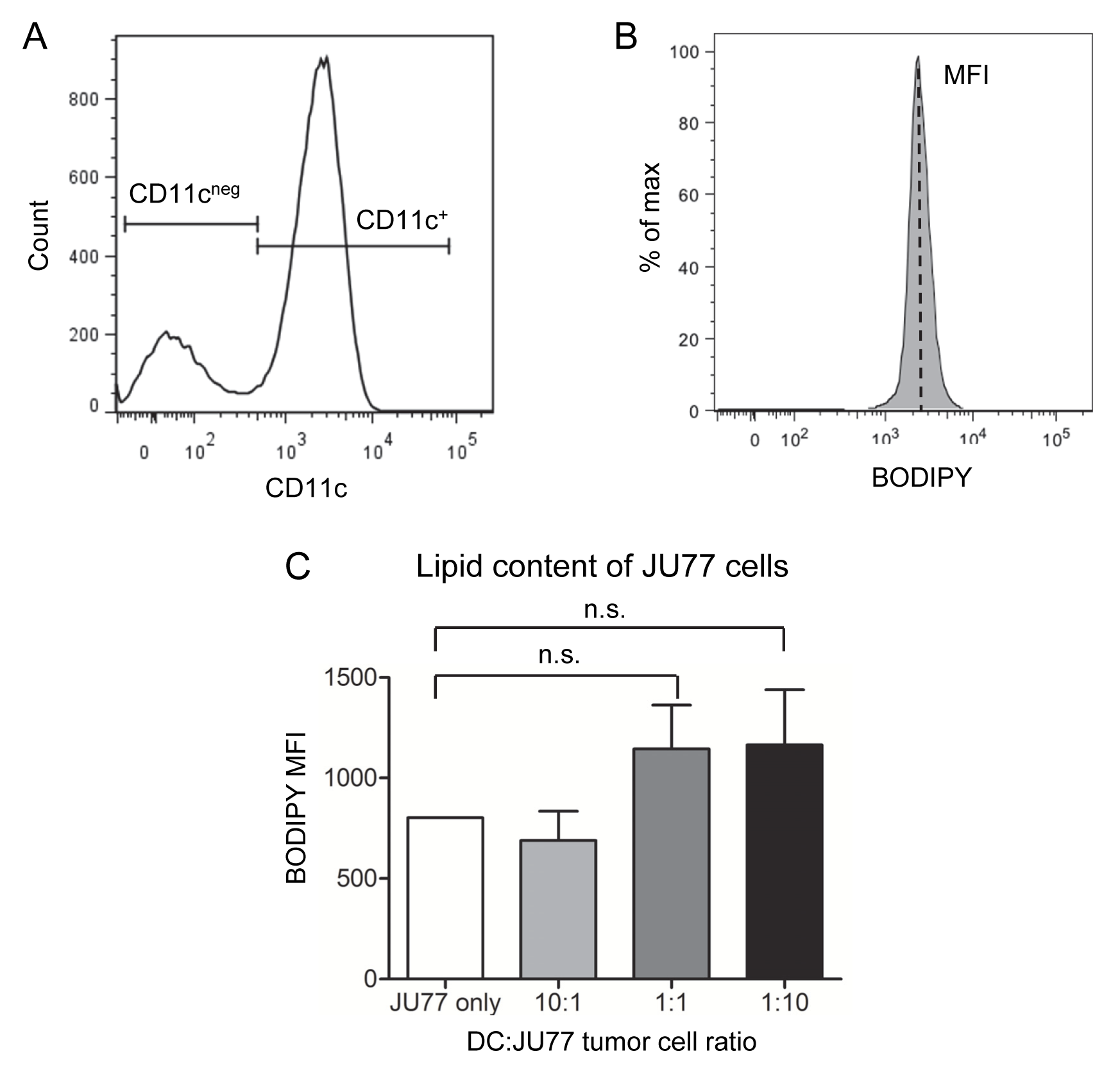

Supplement: S1 Fig — Immature MoDCs were co-cultured with varying ratios of JU77 cells as described in Fig 1A, and then analyzed using flow cytometry for CD11c expression and lipid content. JU77 cells were identified as CD11c negative cells (CD11cneg gate; A) and JU77 lipid content was measured using the MFI of BODIPY staining (B). The lipid content of JU77 cells at various DC:JU77 tumor cell ratios is shown (C). Data is from 1 experiment for JU77 only and 7 experiments for DC:JU77 ratios of 10:1, 1:1 and 1:10. (TIF) [file pone.0123563.s001.tif]

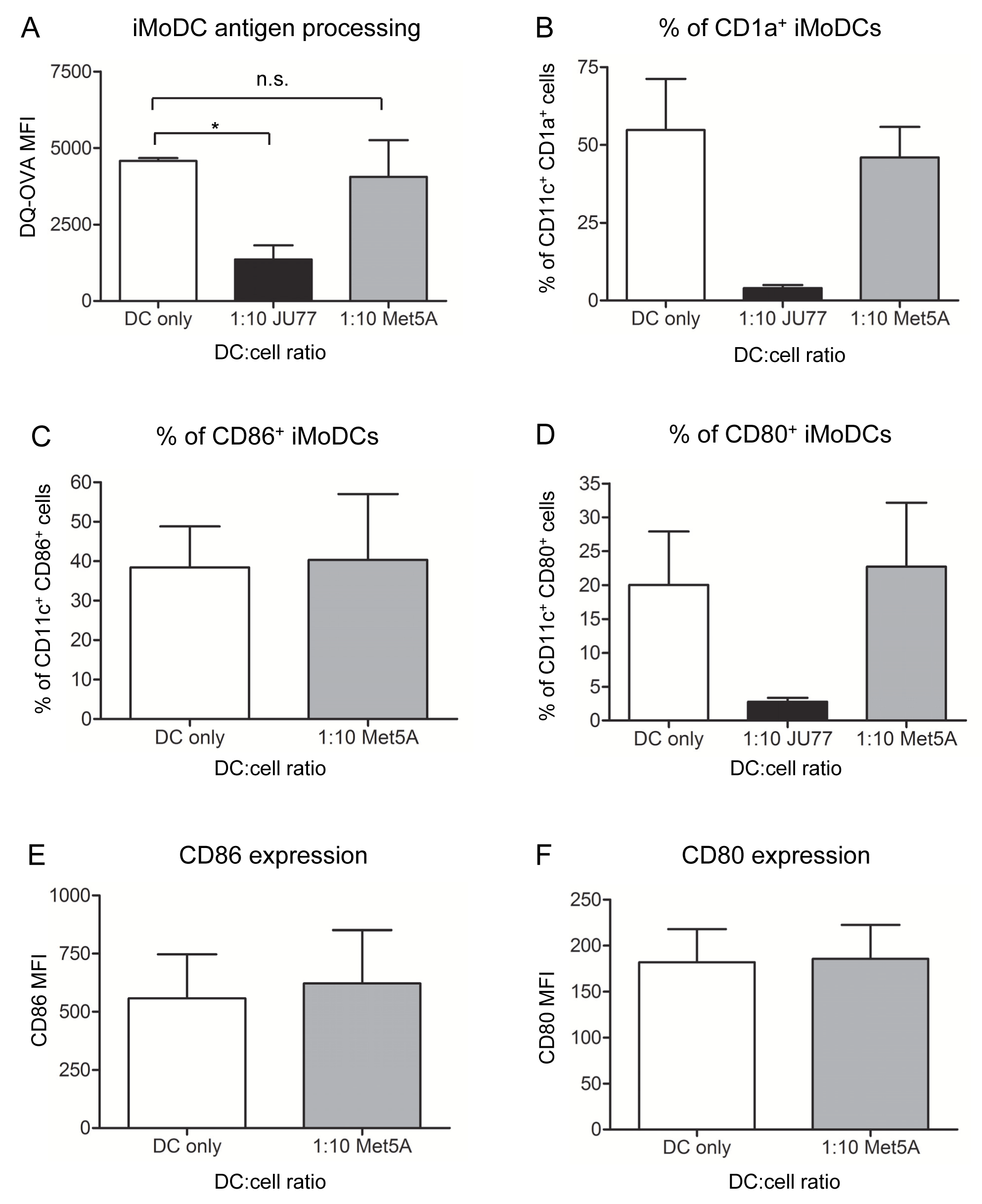

Supplement: S2 Fig — During differentiation, MoDCs were co-cultured with JU77 tumor cells or a control cell line, Met5A, at a ratio of 1 DC:10 JU77 or Met5A cells. Following co-culture, iMoDC antigen processing ability was assessed using the DQ-OVA assay (A). The percent of iMoDCs positive for CD1a (B), CD86 (C) and CD80 (D) were examined. iMoDC expression levels of CD86 (E) and CD80 (F) were also measured using the MFIs of CD86 and CD80 staining. Data is from 3 individuals and is shown as mean ± SEM. * = p < 0.05. (TIF) [file pone.0123563.s002.tif]

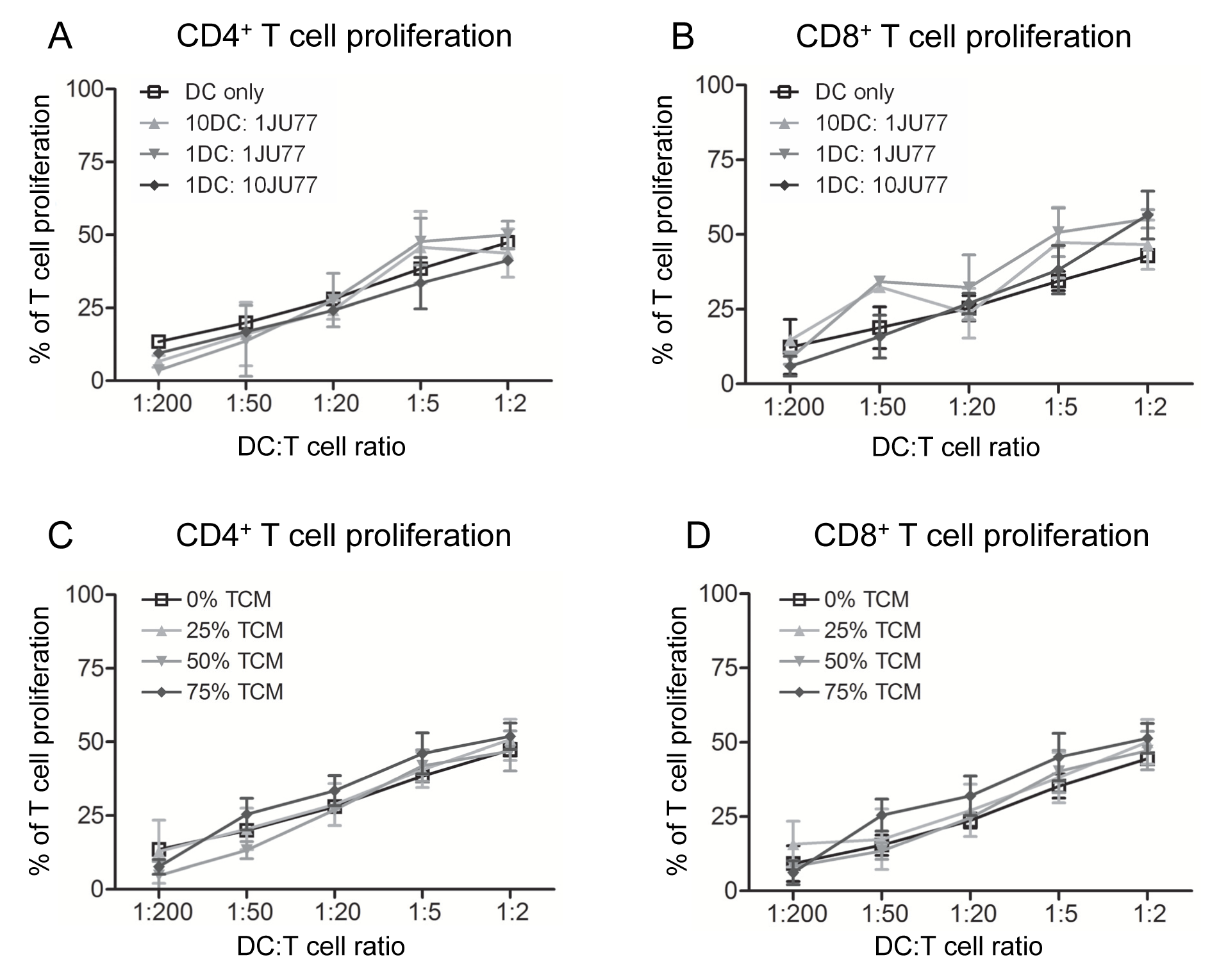

Supplement: S3 Fig — Immature MoDCs were co-cultured with varying ratios of JU77 cells during DC differentiation, as described in Fig 1A. Following co-culture, the ability of immature MoDCs to stimulate CD4+ (A) and CD8+ (B) T cell proliferation was assessed using the allogeneic MLR. Immature MoDCs exposed to varying concentrations of JU77 TCM during differentiation (described in Fig 4A) were also assessed for their ability to stimulate CD4+ (C) and CD8+ (D) T cell proliferation. Pooled data is from 3 individuals and shown as mean ± SEM. (TIF) [file pone.0123563.s003.tif]

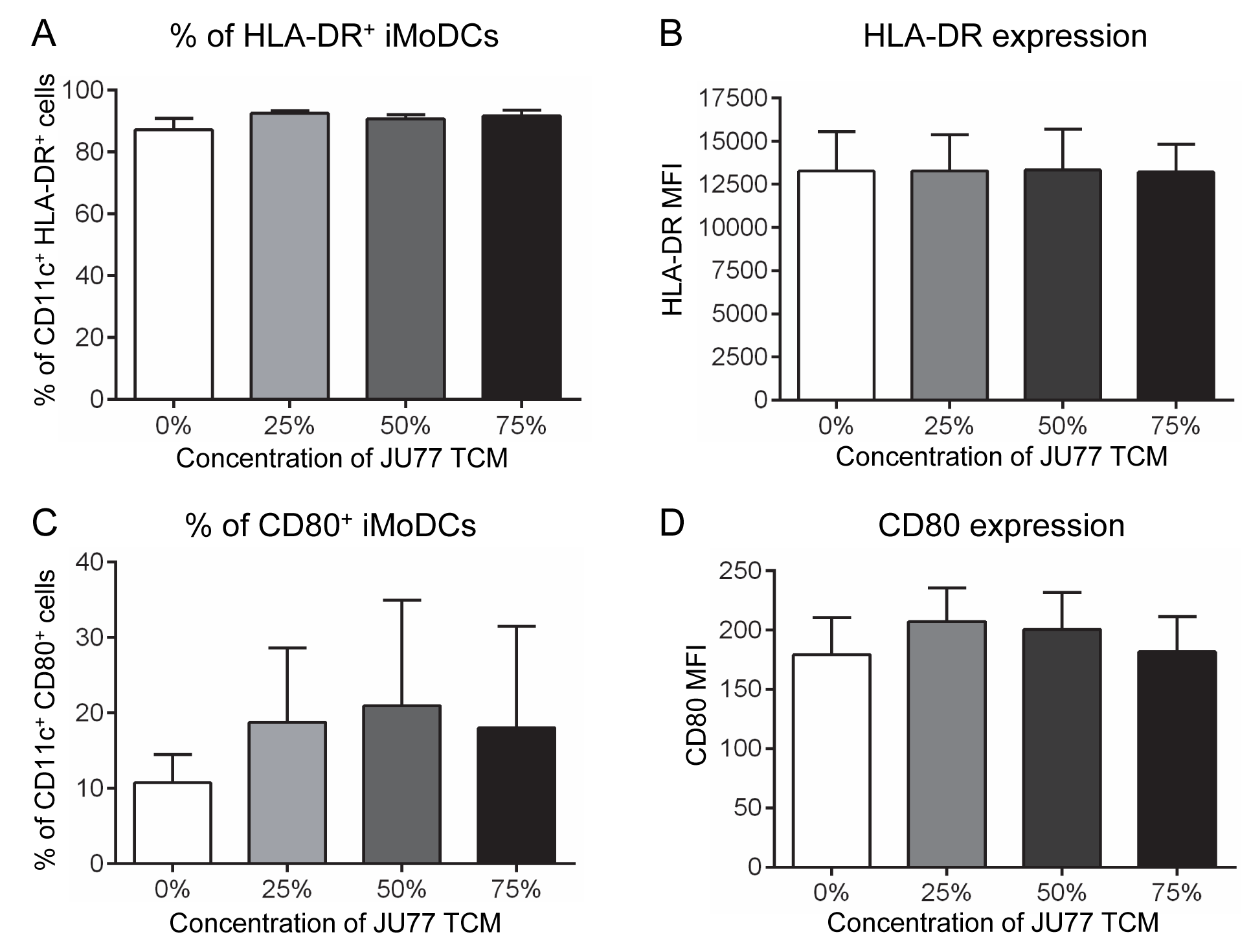

Supplement: S4 Fig — HLA-DR and CD80 expression were measured on iMoDCs cultured in the presence of varying concentrations of JU77 TCM. Pooled data of the percent of iMoDCs expressing HLA-DR (A) and CD80 (C) and surface expression levels (shown as MFIs) of HLA-DR (B) and CD80 (D) on iMoDCs is from 6 individuals and shown as mean ± SEM. (TIF) [file pone.0123563.s004.tif]

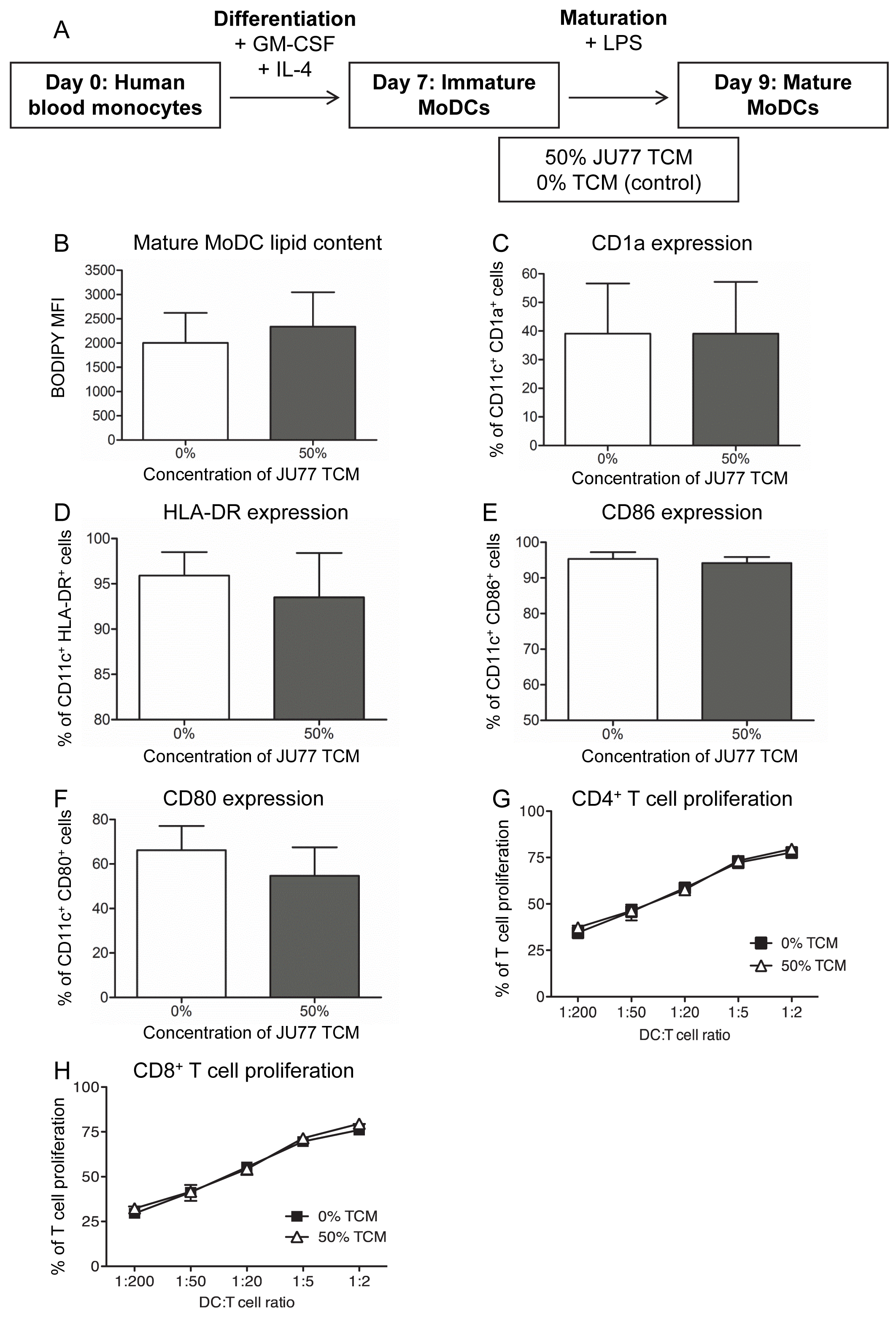

Supplement: S5 Fig — Immature human MoDCs were matured for 2 days using LPS with or without 50% JU77 TCM (A). Lipid levels (shown as MFI; B), and expression of CD1a (C), HLA-DR (D), CD86 (E) and CD80 (F) were measured using flow cytometry. The ability of mature MoDCs to stimulate T cell proliferation was measured using the MLR assay involving CFSE-labelled allogeneic T cells. Mature DCs were co-cultured with varying ratios of T cells, at day 8 non-adherent cells were stained to identify CD4+ and CD8+ T cells. The percent of T cell proliferation was calculated based on the loss of CFSE staining intensity of the parent peak. The ability of mature MoDCs cultured with or without JU77 TCM to stimulate CD4+ (G) and CD8+ (H) T cell proliferation is shown. Pooled data from 4 individuals is shown as mean ± SEM. (TIF) [file pone.0123563.s005.tif]

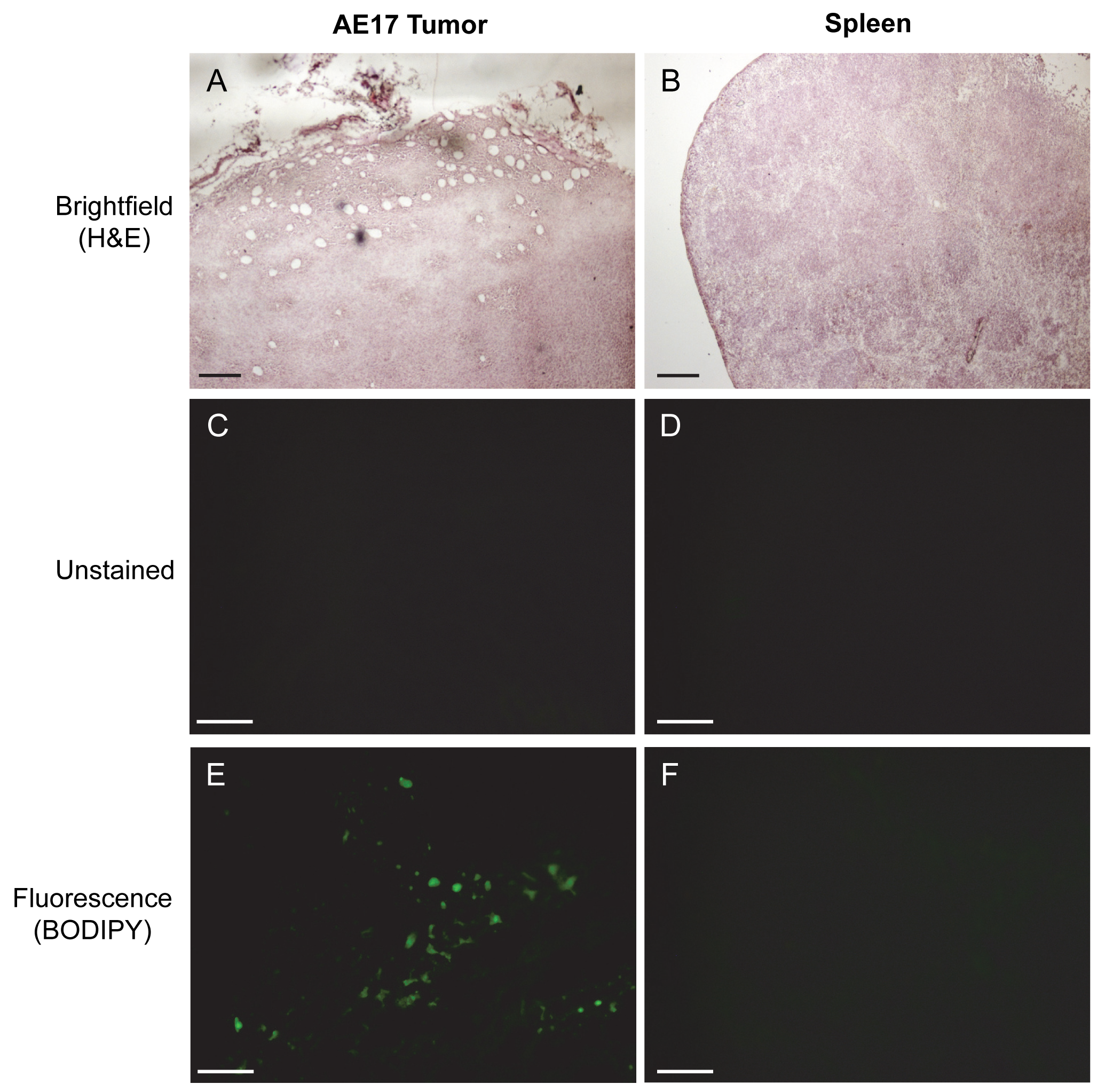

Supplement: S6 Fig — Tumor and spleen sections from AE17 tumor-bearing mice were stained with haematoxylin and eosin (H&E) for general morphology (A and B); scale bars = 200 μm. Unstained sections (C and D) were used as controls for BODIPY-stained tumor and spleen sections (E and F) visualised using fluorescence microscopy; scale bars = 100 μm. Representative images from one experiment are shown. (TIF) [file pone.0123563.s006.tif]

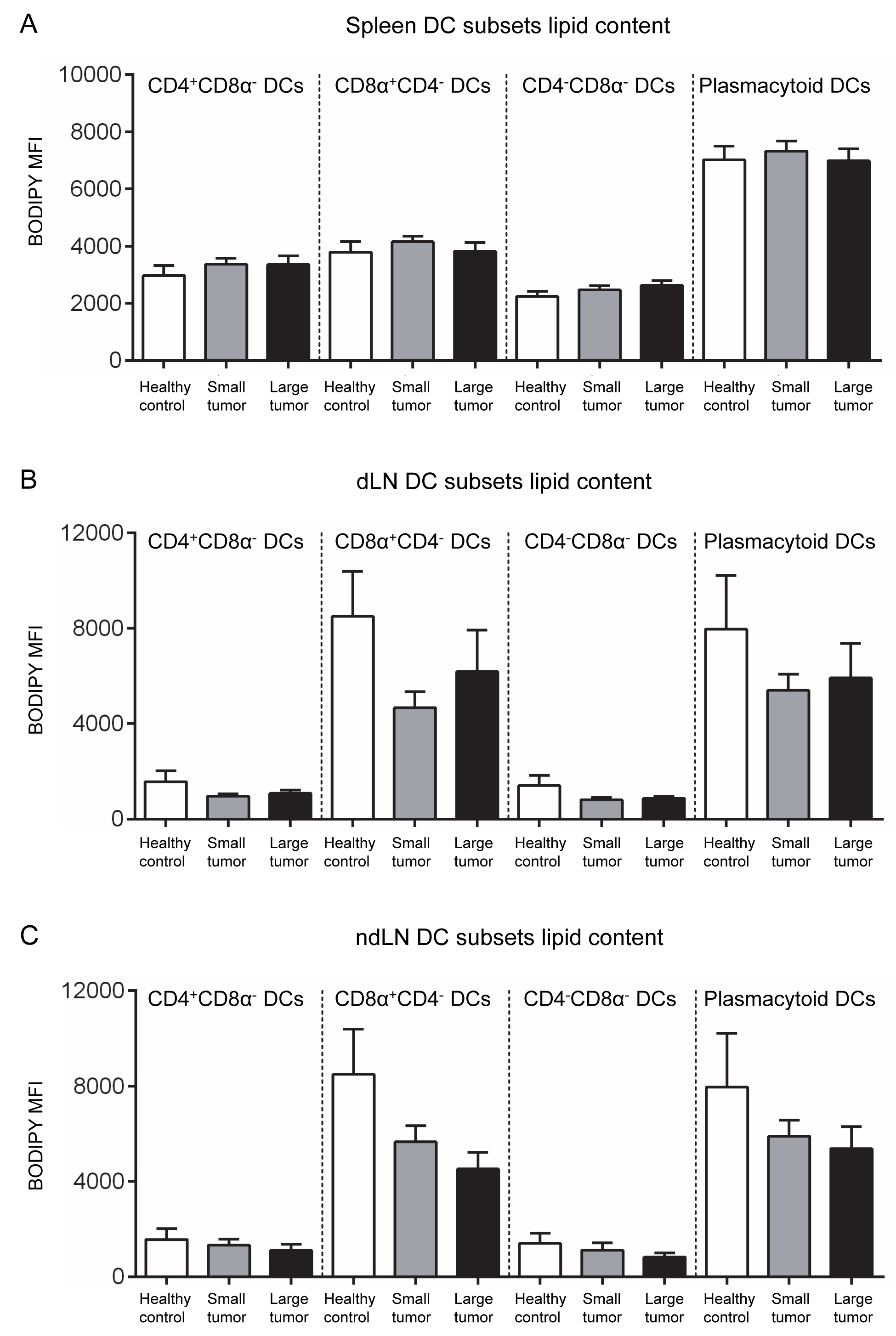

Supplement: S7 Fig — Lipid levels (shown as BODIPY MFIs) of DC subsets were measured in spleens (A), dLNs (B) and ndLNs (C) of tumor-bearing and healthy control mice: n = 18 mice with small tumors, n = 9 mice with large tumors and n = 8 healthy control mice. Pooled data are shown as mean ± SEM. (TIF) [file pone.0123563.s007.tif]

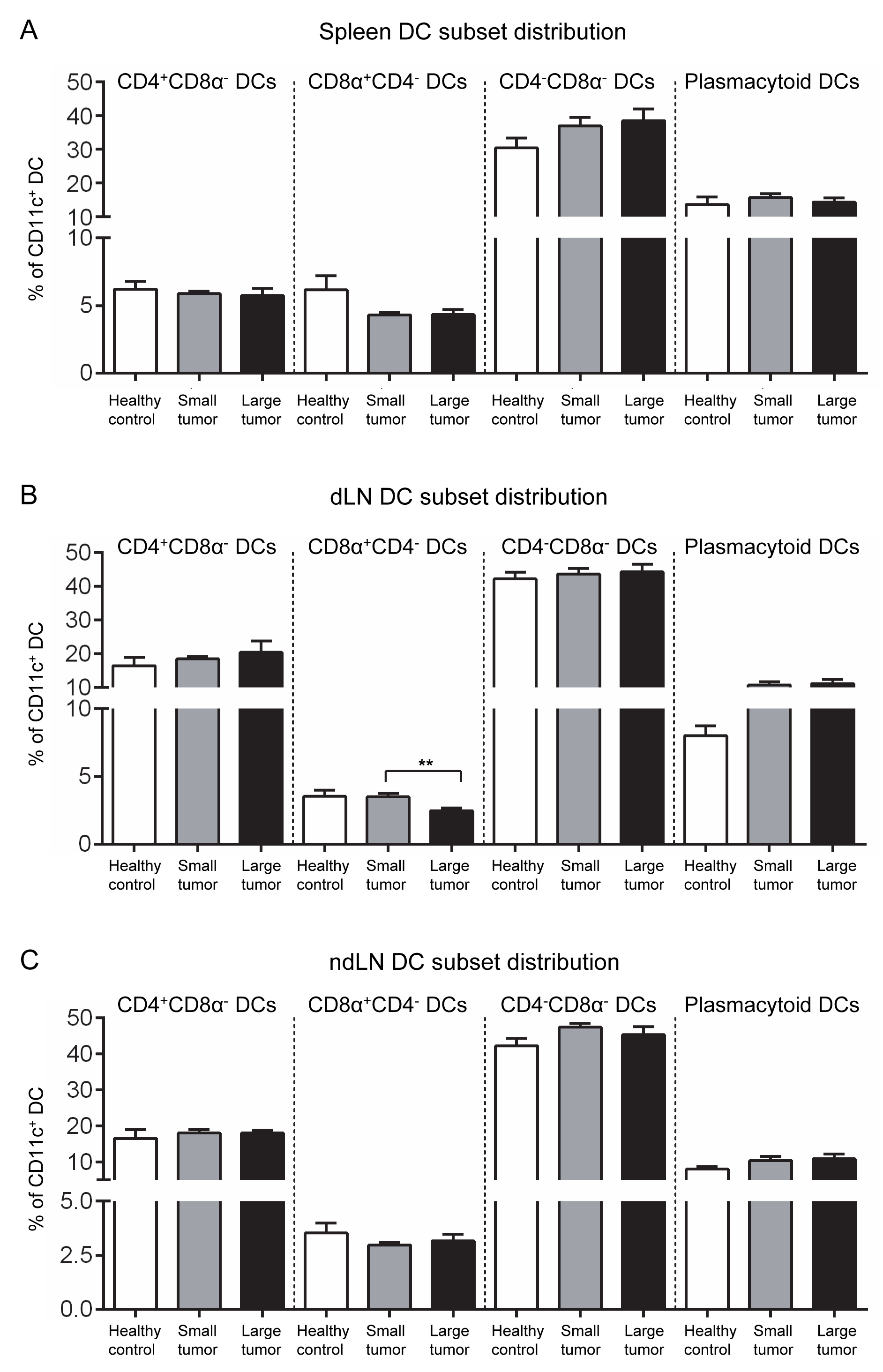

Supplement: S8 Fig — The proportions of DC subsets within spleens (A), dLNs (B) and ndLNs (C) of tumor-bearing and healthy mice were compared: n = 18 mice with small tumors, n = 9 mice with large tumors and n = 8 healthy control mice. Pooled data are shown as mean ± SEM. ** = p < 0.005. (TIF) [file pone.0123563.s008.tif]

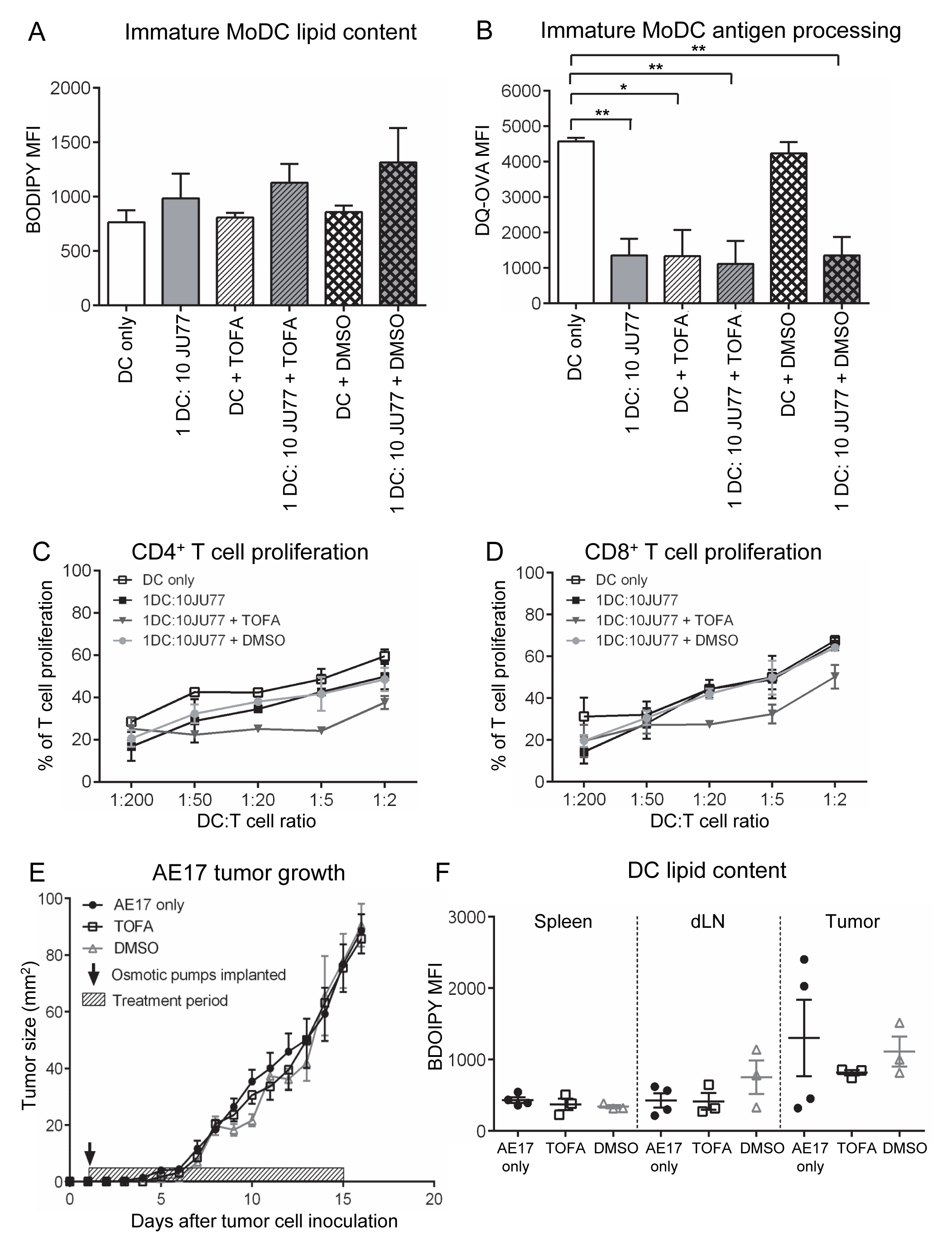

Supplement: S9 Fig — MoDCs were cultured alone or in the presence of JU77 tumor cells (1 DC: 10 JU77 cells). 5 μg/ml TOFA or DMSO was added to MoDC cultures for days 4–7. On day 7, MoDC lipid content (A) and antigen processing capacity (B) were assessed. The ability of MoDCs to stimulate CD4+ T cell (C) and CD8+ T cell (D) proliferation was measured using an MLR assay. Mice inoculated with AE17 tumor cells were left untreated (AE17 only) or implanted with osmotic pumps containing TOFA or DMSO on day 1 following tumor inoculation (black arrow; E). Mice received a dose of 1 μg TOFA/hour for 14 days; the treatment period is indicated by the shaded bar in (E). Tumor size (in mm2) was measured daily (E). DC lipid content in spleens, dLNs and tumors of untreated, TOFA- or DMSO-treated tumor-bearing mice was assessed (F). All data are shown as mean ± SEM. Data in (A)–(D) are pooled from 3 individuals. For murine studies shown in (E) and (F), n = 5 mice/group. Representative data are shown in (F). * = p < 0.05; ** = p < 0.005. (TIF) [file pone.0123563.s009.tif]
